# Supplementary material for: Ion transfer mechanisms in Mrp-type antiporters from high resolution cryoEM and molecular dynamics simulations
Source: Nat Commun. 2022 Oct 14;13:6091. doi: 10.1038/s41467-022-33640-y (PMC9568556; doi:10.1038/s41467-022-33640-y)
Supplement: Supplementary file 1 — Supplementary Information [file 41467_2022_33640_MOESM1_ESM.pdf]

Supplementary Information for

Ion transfer mechanisms in Mrp-type antiporters from high resolution cryoEM and molecular dynamics simulations

Yongchan Lee[1,2,†], Outi Haapanen[3,†], Anton Altmeyer[4,5], Werner Kühlbrandt[1], Vivek Sharma[3,6,*] and Volker Zickermann[4,5,*]

*Corresponding author. Email

vivek.sharma@helsinki.fi

Zickermann@med.uni-frankfurt.de

This PDF file includes:

Supplementary Note

Supplementary Figs. 1 to 12

Supplementary Tables 1 to 8

Supplementary References

Supplementary Note

Coordination of internal water molecules by conserved polar and titratable residues

In MrpA, conserved polar and protonatable residues (Fig. 2, Supplementary Fig. 3, Supplementary Fig. 6, Supplementary Table 4) form a contiguous network extending from the putative proton entry site at Lys408^{MrpA}/Glu409^{MrpA} to His248^{MrpA} in the *A* conformation. Water molecules W72 – W78 are arranged around the strictly conserved Lys408^{MrpA}. The ligation of the water molecules indicates that Gln309^{MrpA} and Tyr447^{MrpA} and several serine and threonine residues play a crucial role, e.g. water molecule W72 is within hydrogen bonding distance of the highly conserved Ser308^{MrpA} and Tyr447^{MrpA} and moderately conserved Gln309^{MrpA} and Thr444^{MrpA}.

From His248^{MrpA} a hydrophilic connection to the cytosolic side is formed by strictly conserved Tyr101^{MrpA}, Ser244^{MrpA}, Lys299^{MrpA} and highly conserved residues Thr241^{MrpA} and Asp297^{MrpA} (Fig. 2). Tyr101^{MrpA} and Thr241^{MrpA} are within hydrogen bonding distance of W70 and W71. Ser244^{MrpA} binds water W69. Note that residues corresponding to Ser244^{MrpA} also bind a water molecule in respiratory complex I [1,2].

His248 in the *B* conformation is associated with a network of polar residues that connects to the strictly conserved Glu140^{MrpA}/Lys223^{MrpA} pair of MrpA. Water W68 is coordinated by the strictly conserved residues Ser146^{MrpA}, Thr170^{MrpA} and non-conserved Ser147^{MrpA}. The adjacent water molecules W66 and W67 are mainly bound by Glu140^{MrpA} and the highly conserved residues Ser143^{MrpA} and Thr222^{MrpA}. Interestingly, the indole NH moiety of the strictly conserved Trp139^{MrpA} coordinates both water molecules.

At the interface of MrpA and MrpD, water W65 is bound toTyr136^{MrpA} and the strictly conserved Lys392^{MrpD}. In MrpD, we modelled six water molecules (W59 – W64) between Lys392^{MrpD} and the highly conserved His332^{MrpD}. The water molecules W59 – W64 are further coordinated by Gln307^{MrpD}, highly conserved His303^{MrpD} and several polar residues of which Tyr329^{MrpD} is highly conserved. Following the hydrated region towards the core of MrpD, Lys250^{MrpD}, His333^{MrpD} and strictly conserved Lys337^{MrpD} form an arrangement that is highly similar to the His349^{MrpA}/Lys254^{MrpA}/Lys353^{MrpA} triad in MrpA described above. Interestingly, the antiporter from *A. flavithermus* shows a different set of residues in this region of the MrpD subunit. His303^{MrpD}, Gln307^{MrpD} and His333^{MrpD} are replaced by Asn303^{MrpD}, Ala307^{MrpD} and Asp333^{MrpD}, respectively. An extensive analysis of sequence alignments showed that the presence of a Gln or Asn at position 303 is strictly linked with the absence of a Gln at position 307 and the presence of an Asp at position 333 (Supplementary Fig. 7).

In contrast to MrpA, two water molecules (W56 and W57) are located in the center of MrpD, coordinated by the strictly conserved residues Lys250^{MrpD} and Tyr233^{MrpD} and highly conserved Thr249^{MrpD}. A pathway from the center of MrpD to the cytosol is expected but not obvious in the structure. We have proposed that in antiporter-like complex I subunits, conserved residues at the end of TMH10 mark the entrance of a proton channel that can be closed by a conserved phenylalanine residue in TMH11 [2]. In Mrp, the corresponding residues at the putative channel entry, Asp295^{MrpD} and Lys297^{MrpD}, are also conserved and water molecule W58 is bound in close proximity. However, no other water molecules were found between W58 and water molecules in the central axis, which might indicate that the pathway is blocked by the strictly conserved Phe341 in TMH11 (Supplementary Fig. 3) as recently described for complex I [2]. An overlay of ND2, ND4 and MrpD shows that the position of this residue agrees well with the “closed” conformation of the ND4 subunit in complex I (Supplementary Fig. 4).

A hydrated path runs from the center of MrpD to the neighboring MrpC subunit. Tyr233^{MrpD} bridges between the W56/W57 pair and a cluster of five water molecules (W51 – W55). This cluster is coordinated by Gln166^{MrpD} and the highly conserved Tyr162^{MrpD}. The latter residue engages in a hydrogen bond with the strictly conserved Ser143^{MrpD}. Water molecule W50 is bound between Lys219^{MrpD} and Glu137^{MrpD}, the strictly conserved Lys/Glu pair of MrpD, and is further ligated by Ser170^{MrpD}. We modelled Lys219^{MrpD} in two conformations, only one of which allows for a hydrogen bond to the water molecule.

In the neighboring MrpC subunit, TMH2 and 3 are at the center of a highly hydrated area. A cluster of water molecules W32 – W37 at the interface of MrpC and MrpD is coordinated by Glu137^{MrpD} and by moderately conserved Ser36^{MrpC}, His40^{MrpC} (see below), Ser80^{MrpC}, Thr84^{MrpC}. Towards the C-terminal domain of MrpA, a large water cluster (W21 – W31) is bound by polar residues of MrpC, His37^{MrpC} and His40^{MrpC}, as well as Thr690^{MrpA} and strictly conserved Gln683^{MrpA} and Glu687^{MrpA}. A polar residue at position 690 of MrpA is strictly conserved. Residues His37^{MrpC} and His40^{MrpC} in TMH2 were recently described as being critical for sodium binding [3]. We note that neither residue is strictly conserved, but histidine or asparagine are the only residues allowed at position 40. A string of water molecules W16 – W20 connects TMH18 of MrpA with the highly conserved Asp38^{MrpF}. Residue Ser75^{MrpF} was modelled in two conformations one of which binds water W16. Two clusters of waters (W6 – W15) are located at the interface of MrpF and MrpG. The larger cluster is arranged around Asp38^{MrpF} and further binding interactions exist with the adjacent Thr39^{MrpF}, Ser68^{MrpF} and Thr40^{MrpG}, Thr44^{MrpG} and Thr79^{MrpG}. Polar residues at positions 40, 44 and 79 of MrpG are highly conserved. The smaller cluster is coordinated by residues from both subunits but none of them is conserved.

A previous study suggested that sodium entry from the cytoplasm occurs at the interface between MrpG and MrpE [3]. We find water molecules W1 – W5 distributed around the proposed sodium entry site and several of the hydrogen-bonding residues including highly conserved His37^{MrpG}, Thr116^{MrpE}, and strictly conserved Thr113^{MrpE}, His131^{MrpE}. A sodium exit [3] or proton entry [4] site was proposed near the highly conserved residues Asp776^{MrpA} and Glu780^{MrpA} in TMH21 of MrpA. Water molecules W45 and W46 are close to the critical residues and are further coordinated by strictly conserved residues Thr777^{MrpA} and Thr75^{MrpC}. Water molecules W38 – W44 are found in a cavity that may form a pathway to the cytoplasm. They are coordinated by the strictly conserved residues Asp678^{MrpA}, Asn766^{MrpA}, and Asp121^{MrpB}. Asn766^{MrpA} was modelled in two different conformations oriented towards different waters in the hydrated path.

Protein hydration and residue-water hydrogen bonding analysis

Our additional analyses reveal that a change in protonation state of an amino acid changes the hydrogen bonding patterns with the surrounding water molecules. As expected, the charged state of a residue forms a larger number of hydrogen bonds with water molecules, whereas fewer hydrogen bonds are observed with charge neutral states (Supplementary Fig. 10). This is also observed by plotting the radial distribution function of water molecules around the residue, where its charged state shows a clear sharp peak relative to its neutral state (Supplementary Fig. 10). We find that the internal hydration of the protein stabilizes rapidly (see also [5]) in both cases when the protein is simulated with structural waters or without them (Fig. 4a and Supplementary Fig. 9e). Our data also reveal that the internal hydration of the protein resembles the structural water content more when simulations are performed in the P state (Fig. 4a and Supplementary Fig. 9, panels a-d, see methods). In the S state simulations, additional water molecules diffuse into the protein interior because of the charged states of amino acid residues. Even though we observe close agreement between the structural waters and the water occupancy map from simulations, some non-overlapping areas are observed (Fig. 4a and Supplementary Fig. 9). These are likely due to the differences in time scales of simulations and experiments (see also [6]).

In the simulated time scales, most of the structural water molecules were found to be replaced by bulk water molecules (Supplementary Fig. 9f). However, the water occupancy in internal regions of the protein remain in agreement with the structural water content, when visualized both at 20% (Fig. 4a) and 50% (Supplementary Fig. 9, panels a-d) occupancy level. Scrutinizing hydrogen bonding between water molecules and selected titratable residues, as well as their lifetimes allowed us to estimate residence times of water molecules in their vicinity (Supplementary Table 8). This also led to the classification of amino acid residues (see Supplementary Table 8). Titratable residues that face

bulk or near-bulk like situations do not show a difference in hydrogen bonding lifetimes when the protonation state changes. Interestingly, a putative sodium binding site acidic residue (Glu780^{MrpA}) consistently show nanosecond long residence times of water molecules either in its charge neutral or deprotonated state. This is because in its anionic state, it strongly binds a sodium ion, which replaces the role of its protonated sidechain. In addition, we find that the buried residues with conserved structural water next to them show relatively longer hydrogen bonding residence times of water molecules (Supplementary Table 8).

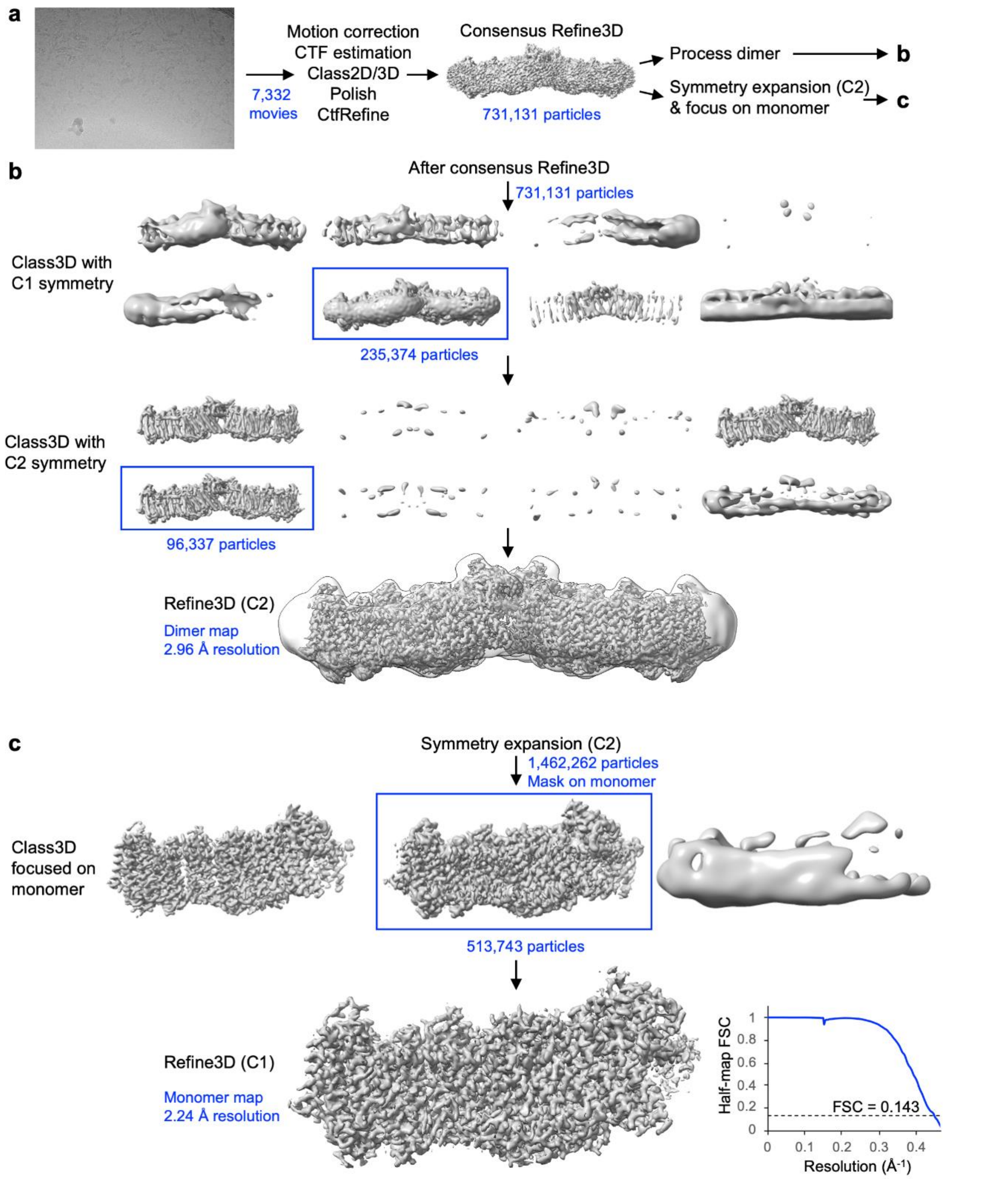

Supplementary Fig. 1.

Workflow of single-particle data processing for *B. pseudofirmus* Mrp. (a) Initial stages of data processing before particles were subjected to different processing strategies. **(b)** Dimer refinement. In the final round of 3D classification, three meaningful classes were observed, showing different angles between the protomers. The one with an intermediate angle was selected for final refinement, (marked by blue rectangle) and yielded the dimer map with the highest quality. The micelle density, lowpass filtered to 12 Å, is overlaid on the final map to visualize membrane boundaries. **(c)** Monomer refinement.

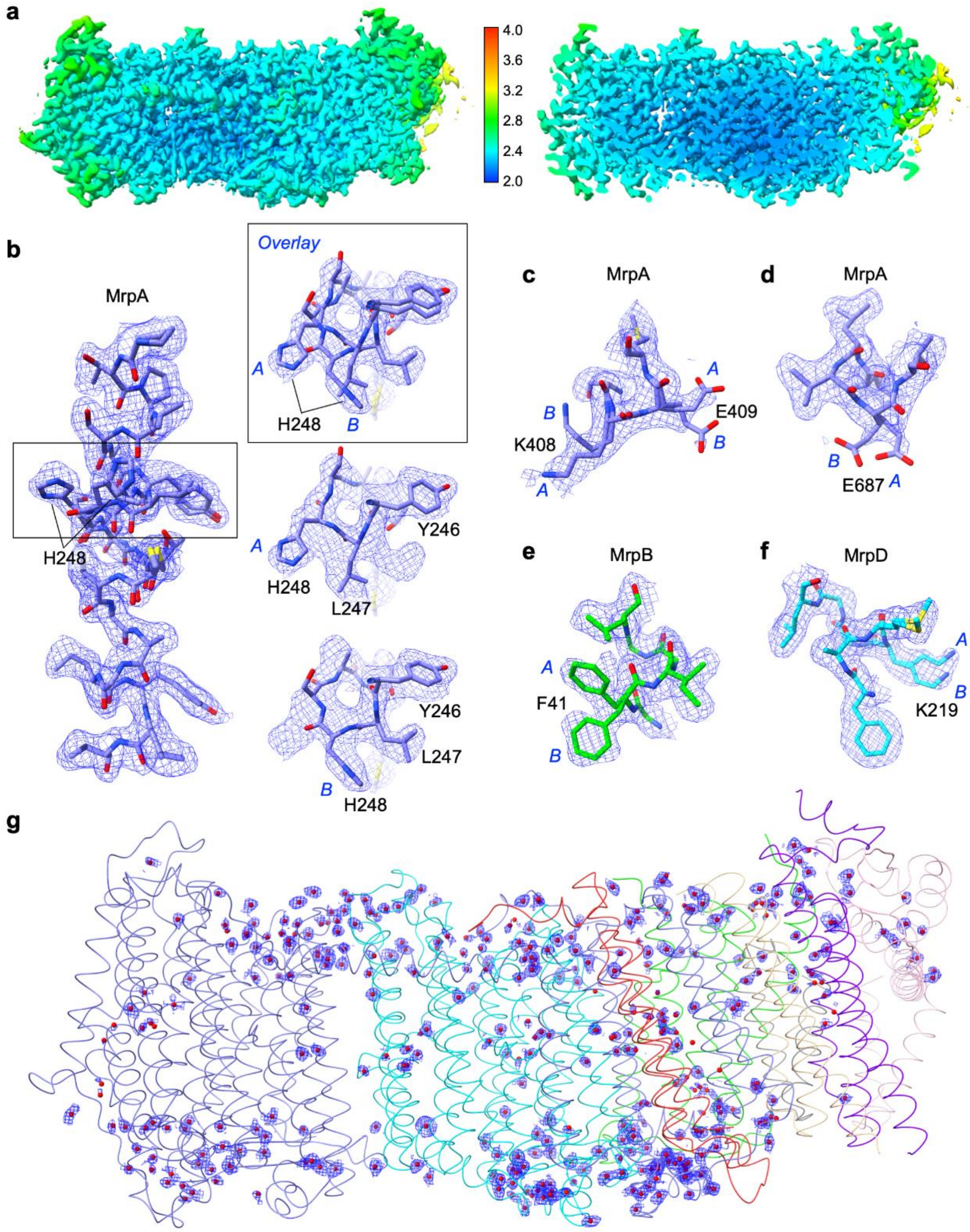

Supplementary Fig. 2.

Local resolution and examples of cryoEM densities. (a) Local resolution map of the *B. pseudofirmus* Mrp monomer**. (b)** Two alternative conformations of residues 246 – 252 in MrpA. Overlaid models from two different views are outlined. Separated models of two conformations are shown below. **(c)** Alternative sidechain conformations of K408 and E409 in MrpA. **(d)** Alternative sidechain conformations of E687 in MrpA. **(e)** Alternative sidechain conformations of F41 in MrpB. **(f)** Alternative sidechain conformations of K219 in MrpD**. (g)** All solvent densities that were modelled as water. Maps are resampled to 0.536 Å/pixel for visualization.

a MrpA-ND5:

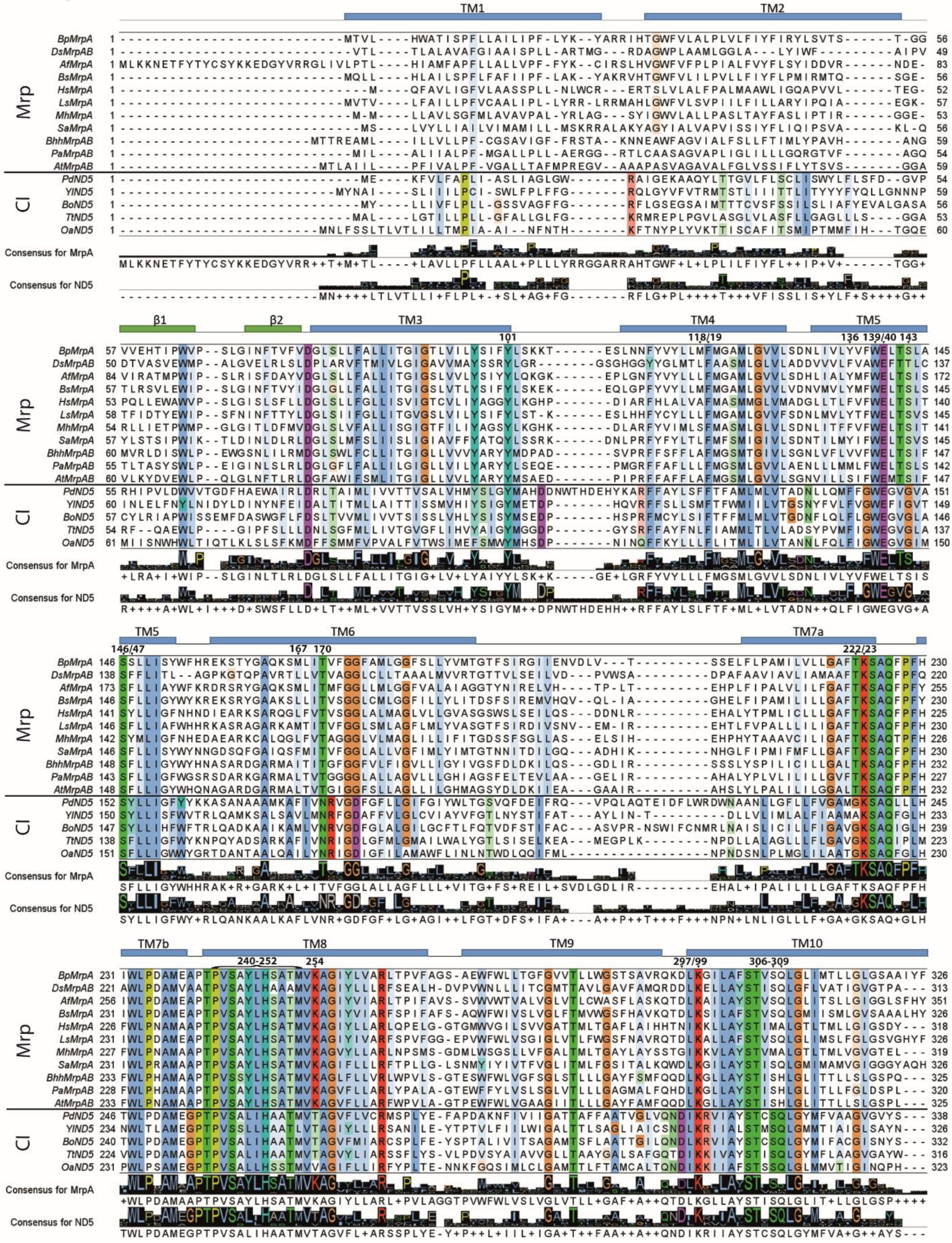

TM1
TM2
TM3
TM4
TM5
TM6
TM7a
TM7b
TM8
TM9
TM10
β1
β2
BpMrpA
DsMrpAB
AfMrpA
BsMrpA
HsMrpA
LsMrpA
MhMrpA
SaMrpA
BhhMrpAB
PaMrpAB
AtMrpAB
PdND5
YlND5
BoND5
TtND5
OaND5
Mrp
CI
Consensus for MrpA
Consensus for ND5

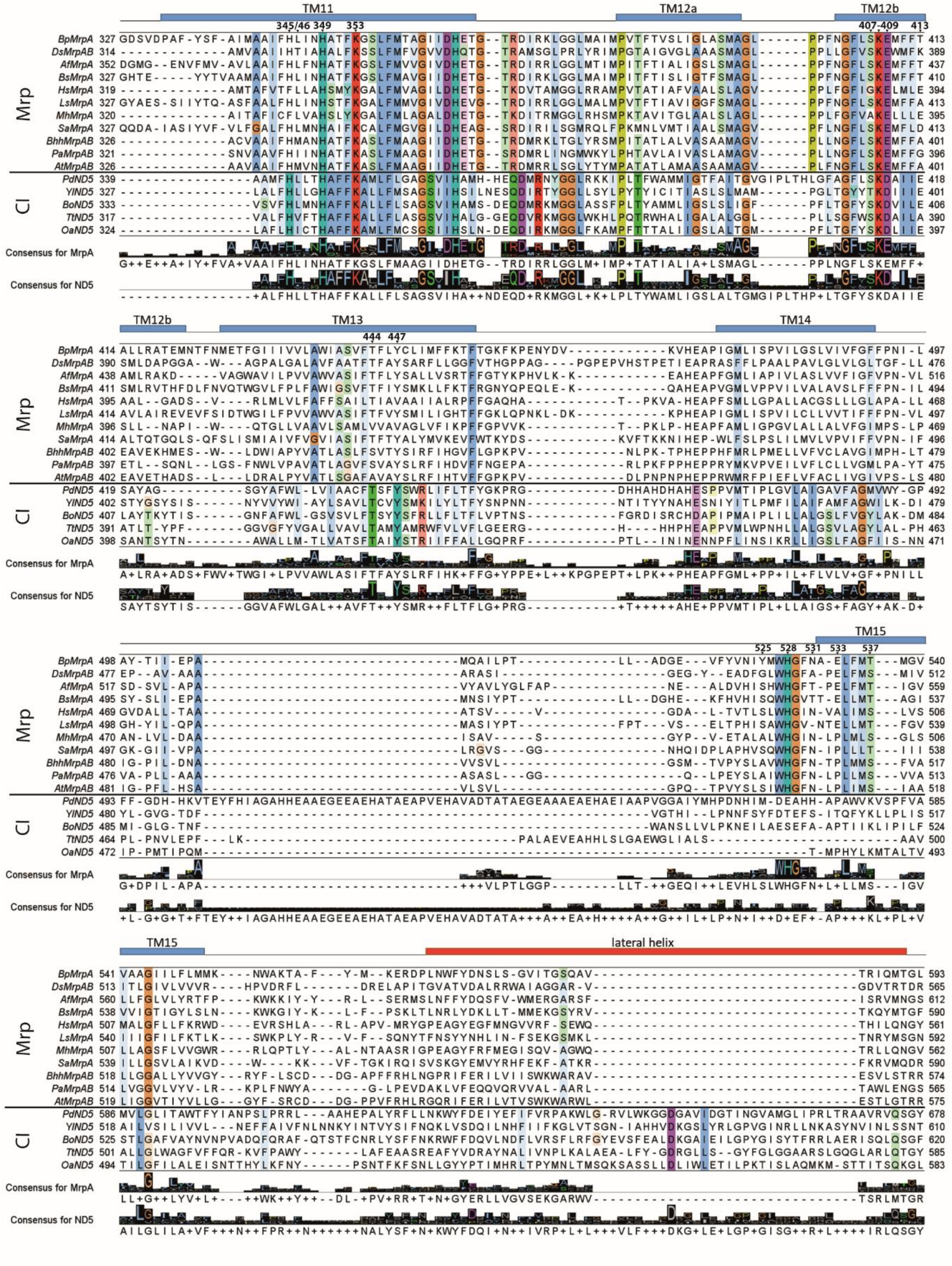
TM11
TM12a
TM12b
TM13
TM14
TM15
lateral helix
Mrp
CI
BpMrpA
DsMrpAB
AfMrpA
BsMrpA
HsMrpA
LsMrpA
MhMrpA
SaMrpA
BhhMrpAB
PaMrpAB
AtMrpAB
PdND5
YlND5
BoND5
TtND5
OaND5
Consensus for MrpA
Consensus for ND5

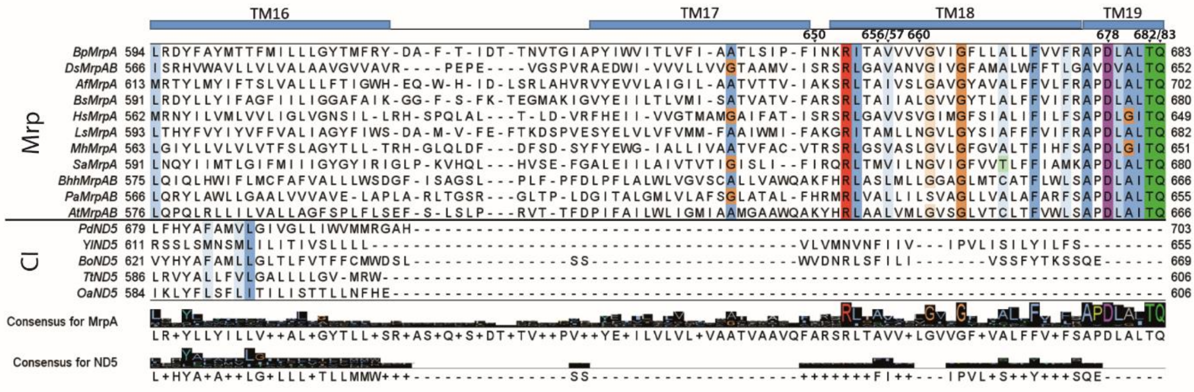
TM16
TM17
TM18
TM19
Mrp
CI
BpMrpA
DsMrpAB
AfMrpA
BsMrpA
HsMrpA
LsMrpA
MhMrpA
SaMrpA
BhhMrpAB
PaMrpAB
AtMrpAB
PdND5
YlND5
BoND5
TtND5
OaND5
Consensus for MrpA
Consensus for ND5

b MrpA-ND6:

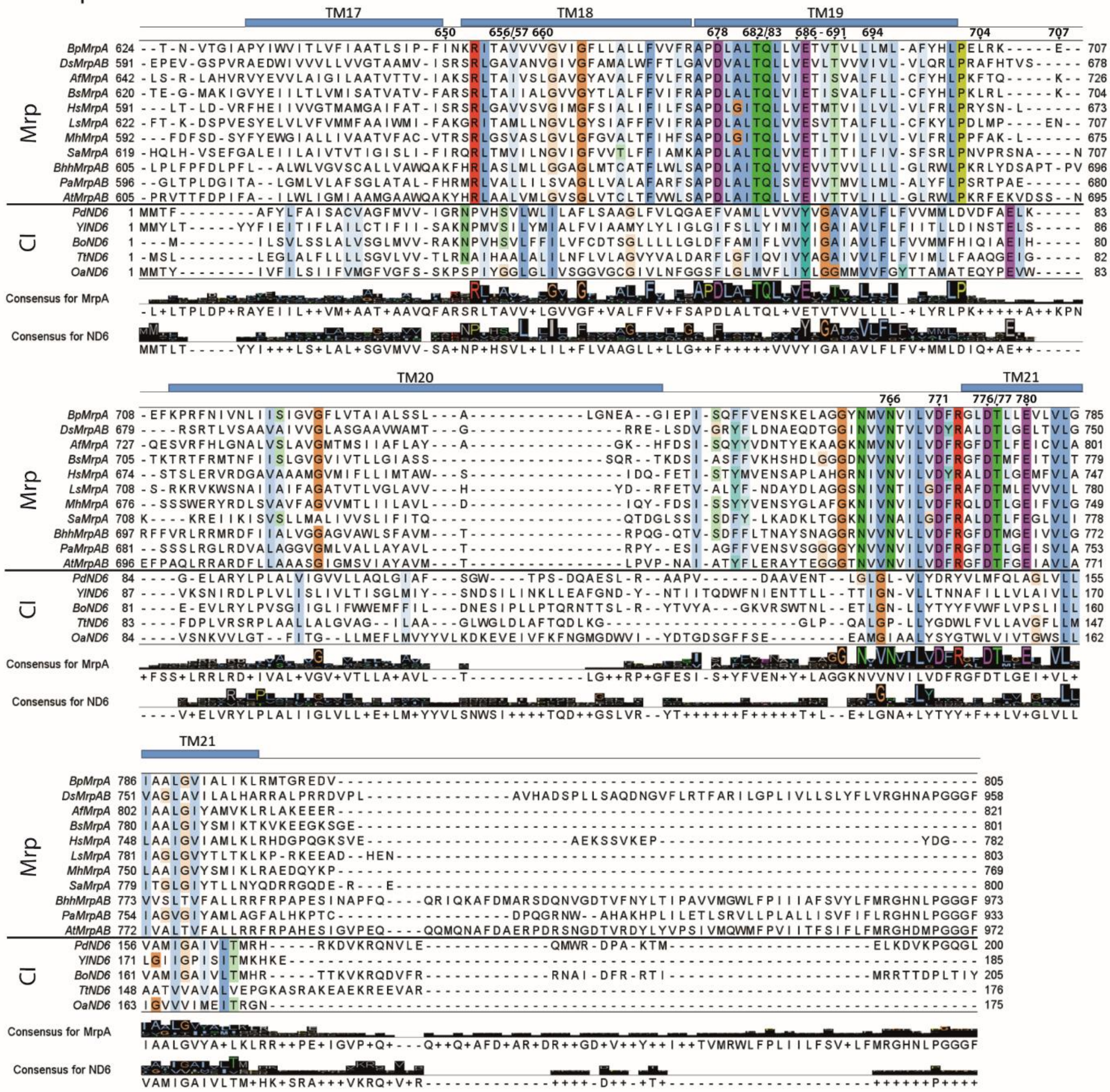
TM17
TM18
TM19
TM20
TM21
Mrp
CI
BpMrpA
DsMrpAB
AfMrpA
BsMrpA
HsMrpA
LsMrpA
MhMrpA
SaMrpA
BhhMrpAB
PaMrpAB
AtMrpAB
PdND6
YlND6
BoND6
TtND6
OaND6
Consensus for MrpA
Consensus for ND6

c MrpB:

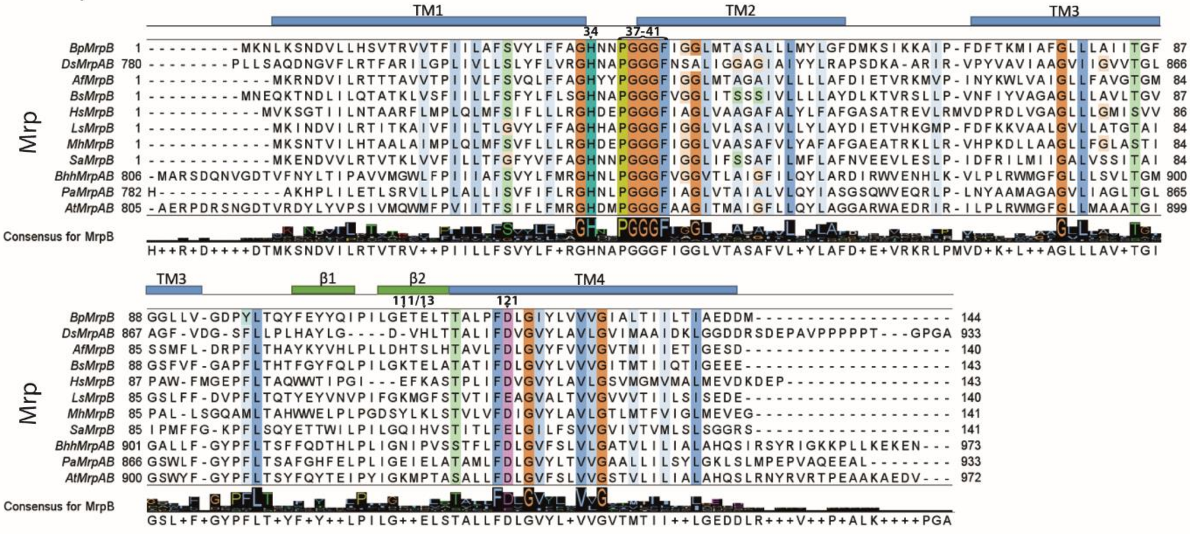

d MrpC-ND4L:

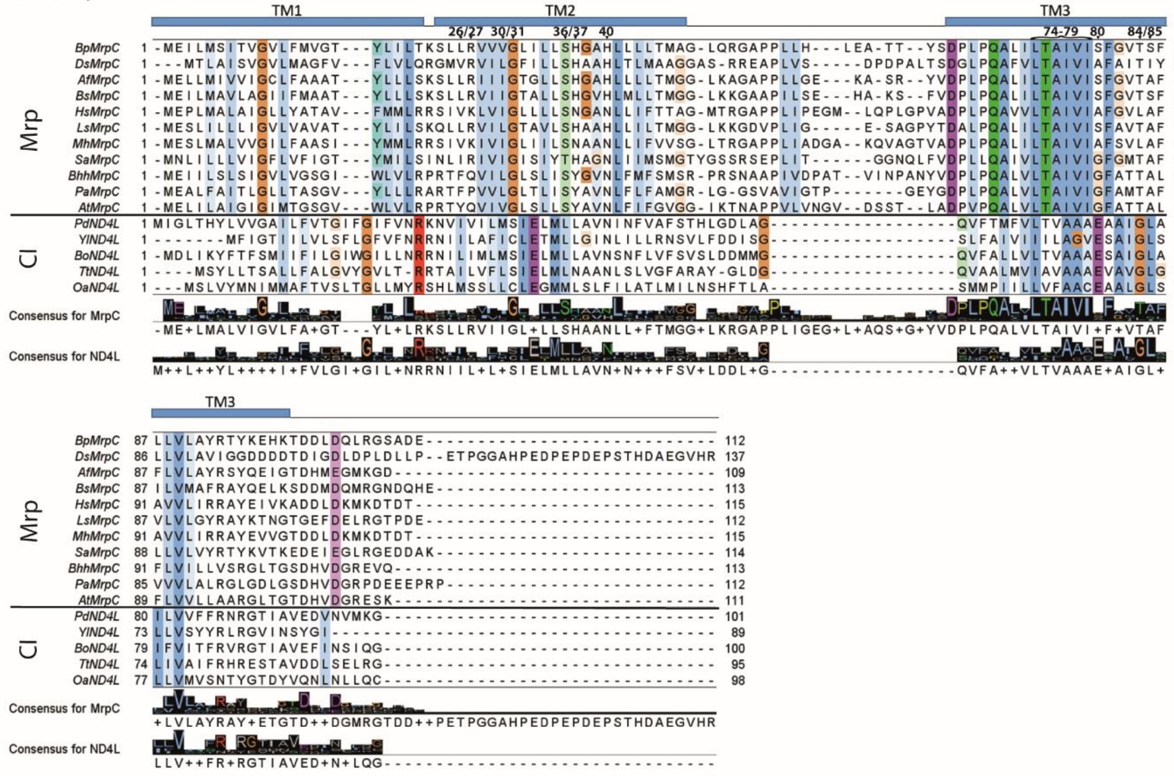

e MrpD-ND2/ND4:

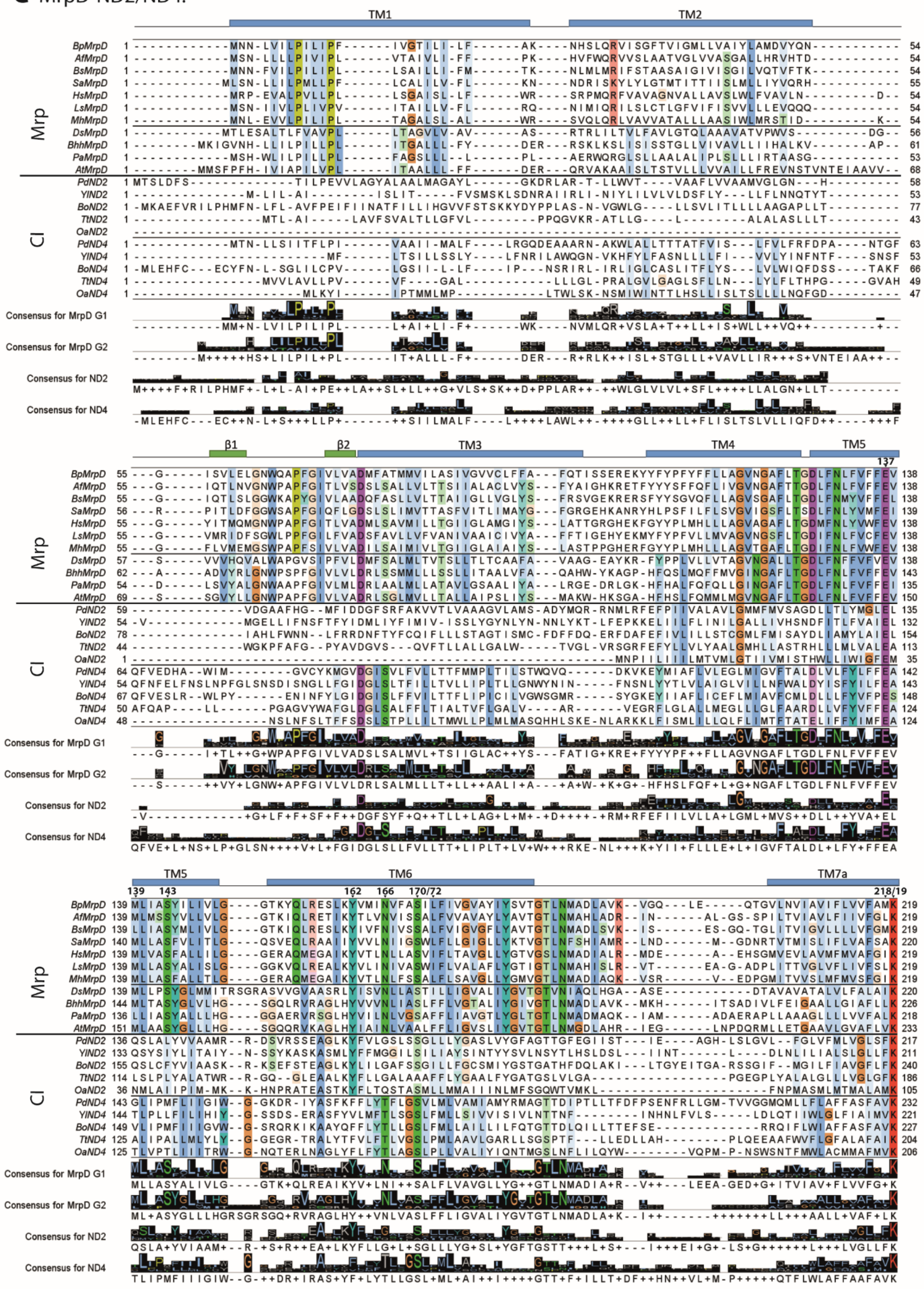

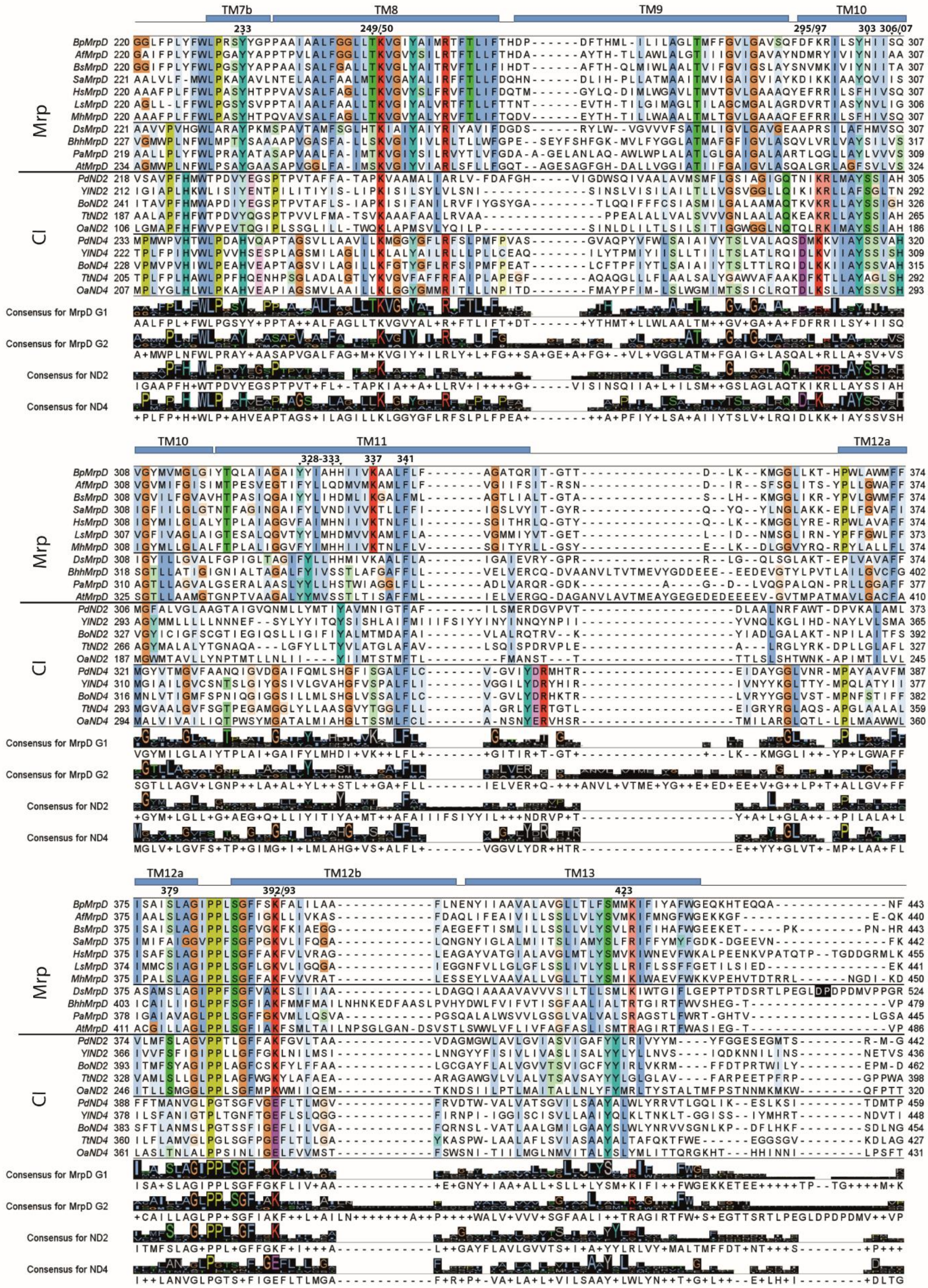

TM7b
TM8
TM9
TM10
TM11
TM12a
TM12b
TM13
Mrp
CI
BpMrpD
AfMrpD
BsMrpD
SaMrpD
HsMrpD
LsMrpD
MhMrpD
DsMrpD
BhhMrpD
PaMrpD
AtMrpD
PdND2
YlND2
BoND2
TtND2
OaND2
PdND4
YlND4
BoND4
TtND4
OaND4
Consensus for MrpD G1
Consensus for MrpD G2
Consensus for ND2
Consensus for ND4

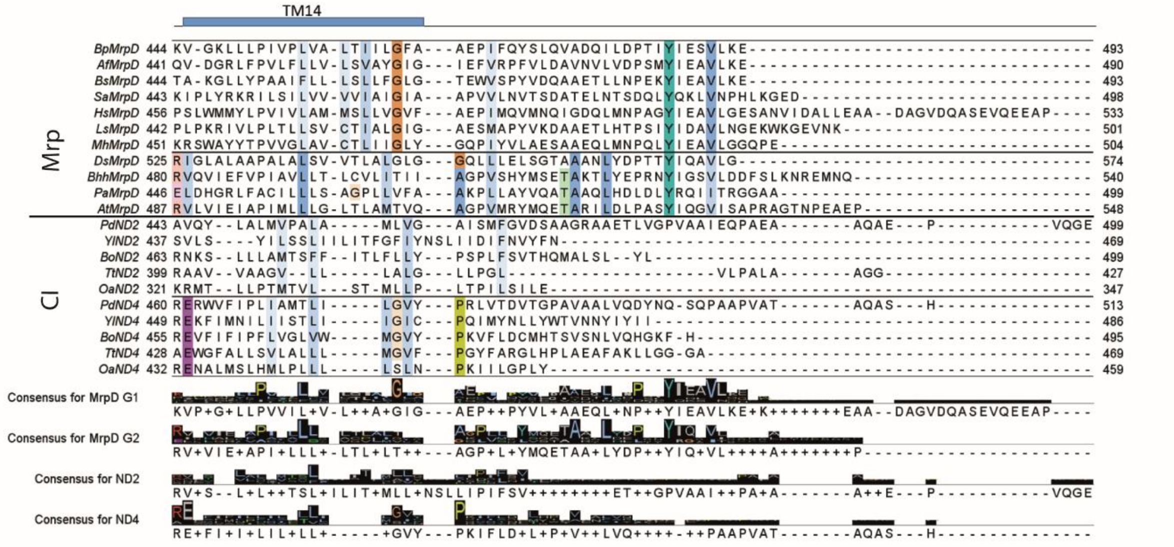
TM14
BpMrpD
AfMrpD
BsMrpD
SaMrpD
HsMrpD
LsMrpD
MhMrpD
DsMrpD
BhhMrpD
PaMrpD
AtMrpD
PdND2
YlND2
BoND2
TtND2
OaND2
PdND4
YlND4
BoND4
TtND4
OaND4
Mrp
CI
Consensus for MrpD G1
Consensus for MrpD G2
Consensus for ND2
Consensus for ND4

f MrpE:

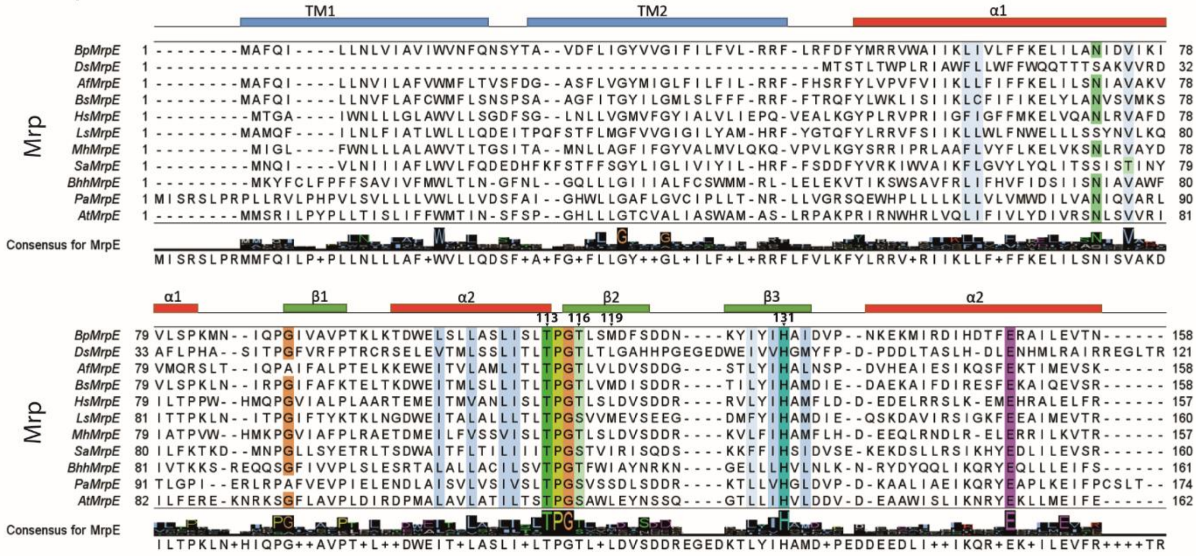
TM1
TM2
α1
β1
α2
β2
β3
BpMrpE
DsMrpE
AfMrpE
BsMrpE
HsMrpE
LsMrpE
MhMrpE
SaMrpE
BhhMrpE
PaMrpE
AtMrpE
Mrp
Consensus for MrpE

g MrpF:

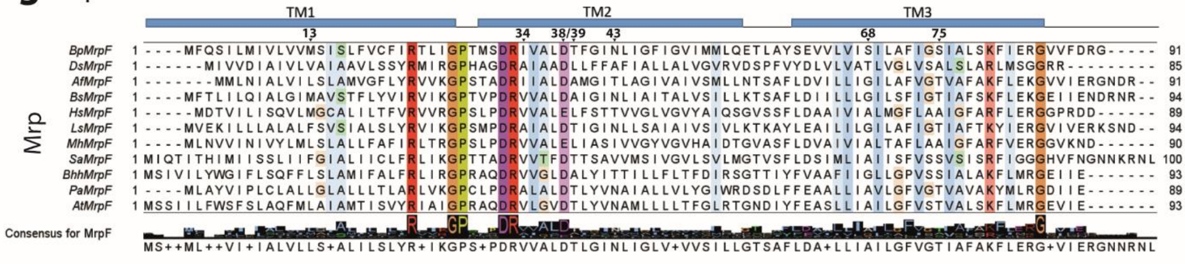
TM1
TM2
TM3
BpMrpF
DsMrpF
AfMrpF
BsMrpF
HsMrpF
LsMrpF
MhMrpF
SaMrpF
BhhMrpF
PaMrpF
AtMrpF
Mrp
Consensus for MrpF

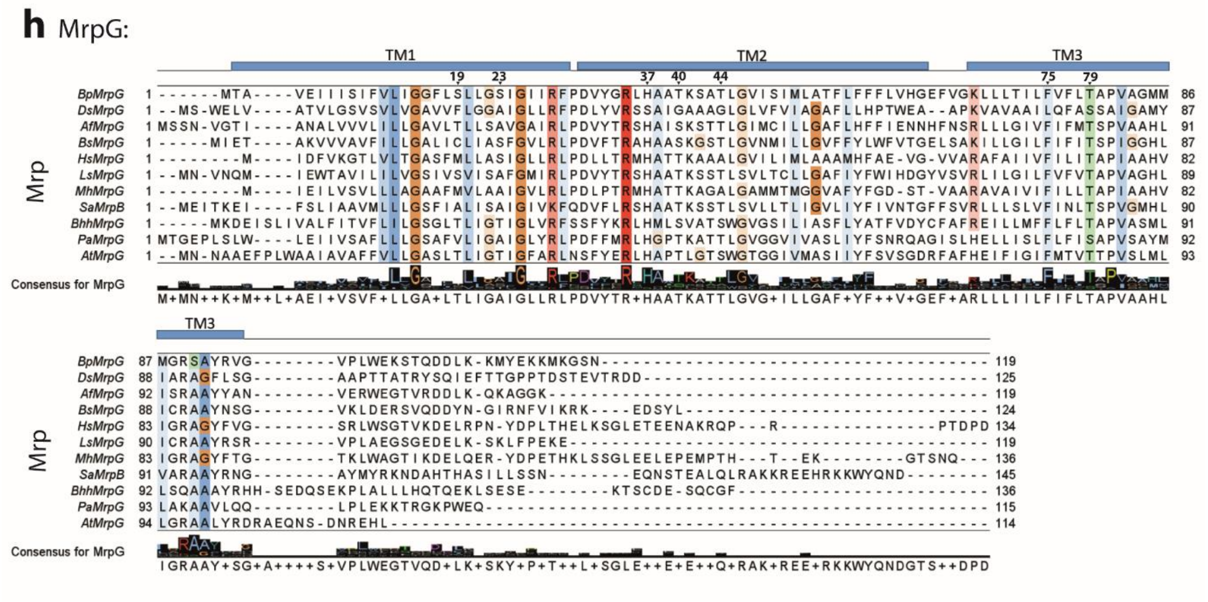

Supplementary Fig. 3.

Sequence alignment of orthologous subunits in Mrp antiporters and respiratory complex I. Alignments include the Mrp antiporter subunits A-G and A'-G from the species *Bacillus pseudofirmus* (BpMrp), *Anoxybacillus flavithermus* (AfMrp), *Bacillus subtilis* (BsMrp), *Staphylococcus aureus* (SaMrp), *Halomonas* sp. (HsMrp), *Lysinibacillus sphaericus* (LsMrp), *Marinobacter hydrocarbonoclasticus* (MhMrp), *Dietzia* sp. (DsMrp), *Bartonella henselae* (BhhMrp), *Pseudomonas aeruginosa* (PaMrp) and *Agrobacterium tumefaciens* (AtMrp), as well as the complex I subunits ND2, ND4, ND5, ND6 and ND4L from the species *Paracoccus denitrificans* (PdND), *Yarrowia lipolytica* (YlND), *Brassica oleracea* (BoND), *Thermus thermophilus* (TtND) and *Ovis aries* (OaND).

The alignments were created using ClustalO [7] with default values in Jalview 2.11.1.4 [8] and are color-coded in the ClustalX [9] preset filtered by 50 % conservation. Selected residues are marked above the alignments with their sequence position in BpMrp. The secondary structure elements of BpMrp subunits are indicated as blue (transmembrane helices: TM), red (helices outside the membrane: α) or green (β-sheets: β) boxes. **(a)** Alignment of subunits MrpA, MrpA' and ND5. **(b)** Alignment of the subunits MrpA, MrpA' and ND6. **(c)** Alignment of subunit MrpB. **(d)** Alignment of subunits MrpC and ND4L. **(e)** Alignment of subunits MrpD, ND2 and ND4. The MrpD sequences are differentiated between group 1 (separate MrpA and MrpB) and group 2 (MrpA' fusion protein) operons. A 68 residue insert between Asp447 and Pro515 in Ds MrpD is not displayed (position highlighted in black). **(f)** Alignment of subunit MrpE. **(g)** Alignment of subunit MrpF. **(h)** Alignment of subunit MrpG.

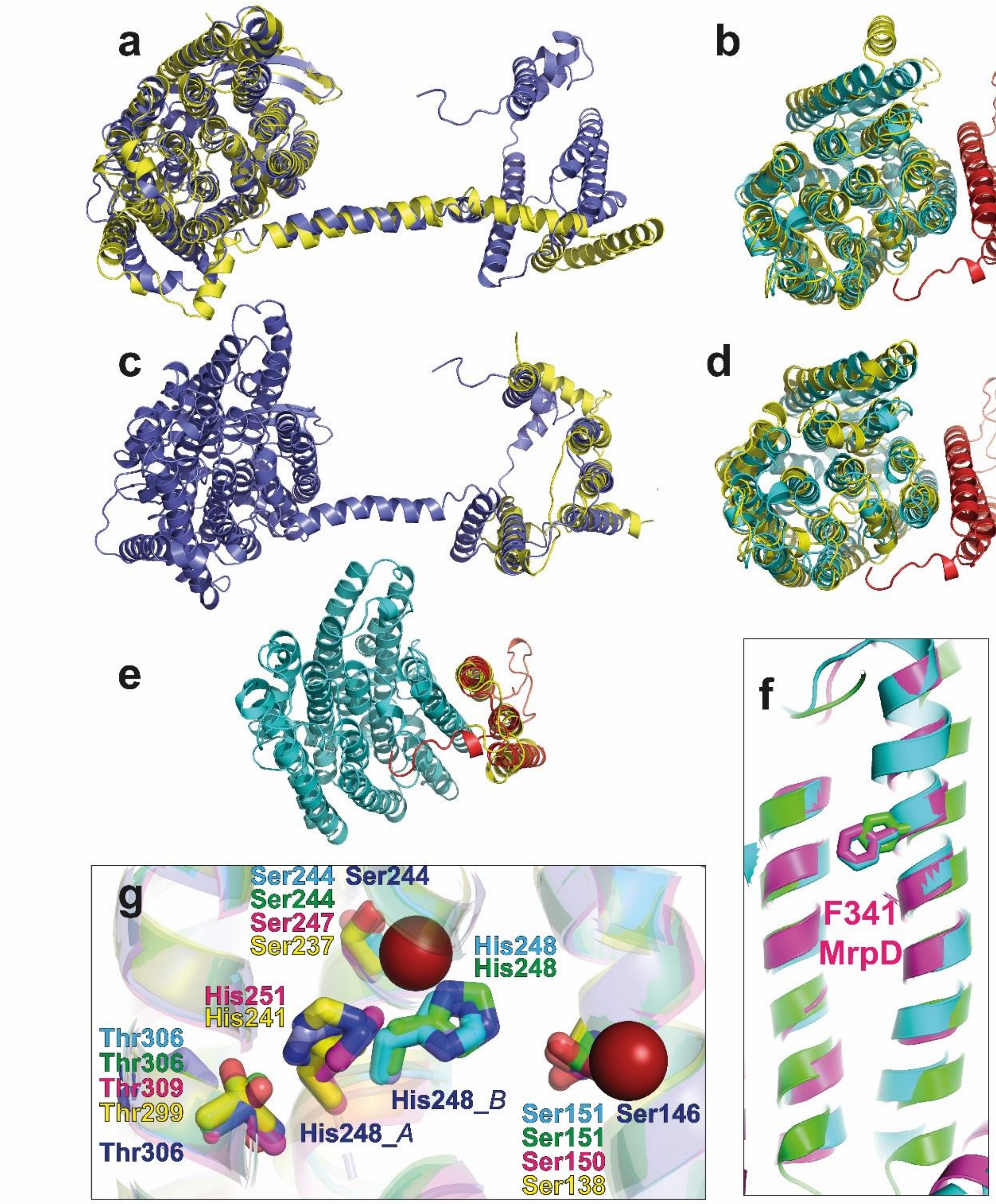

Supplementary Fig. 4.

Structural features of the Mrp antiporter conserved in complex I. Overlay of complex I (*Y. lipolytica*, PDB 7O71, yellow) and Mrp antiporter subunits (color as in Fig. 1), **(a)** MrpA/ND5, **(b)** MrpD/ND4, **(c)** MrpA/ND6, **(d)** MrpD/ND2, **(e)** MrpC/ND4L. **(f)** The position of a highly conserved phenylalanine residue opens or closes a proton pathway in subunits ND2 (green, open) and ND4 (cyan, closed); the position of the corresponding residue Phe341 in MrpD (magenta) matches the closed conformation. **(g)** In complex I structures, residues corresponding to $His248^{MrpA}$ (dark blue, compare Fig. 2) are either in the *A* (e.g. *T. thermophilus* PDB 6I1P (yellow), *Y. lipolytica* PDB 7O71 (magenta)) or *B* conformation (e.g. human PDB 5XTD (cyan), sheep PDB 6ZKA (green)); residues corresponding to $Ser146^{MrpA}$, $Ser244^{MrpA}$, and $Thr306^{MrpA}$ (compare Fig. 2) are strictly conserved in complex I and form connections to three pathways for protons.

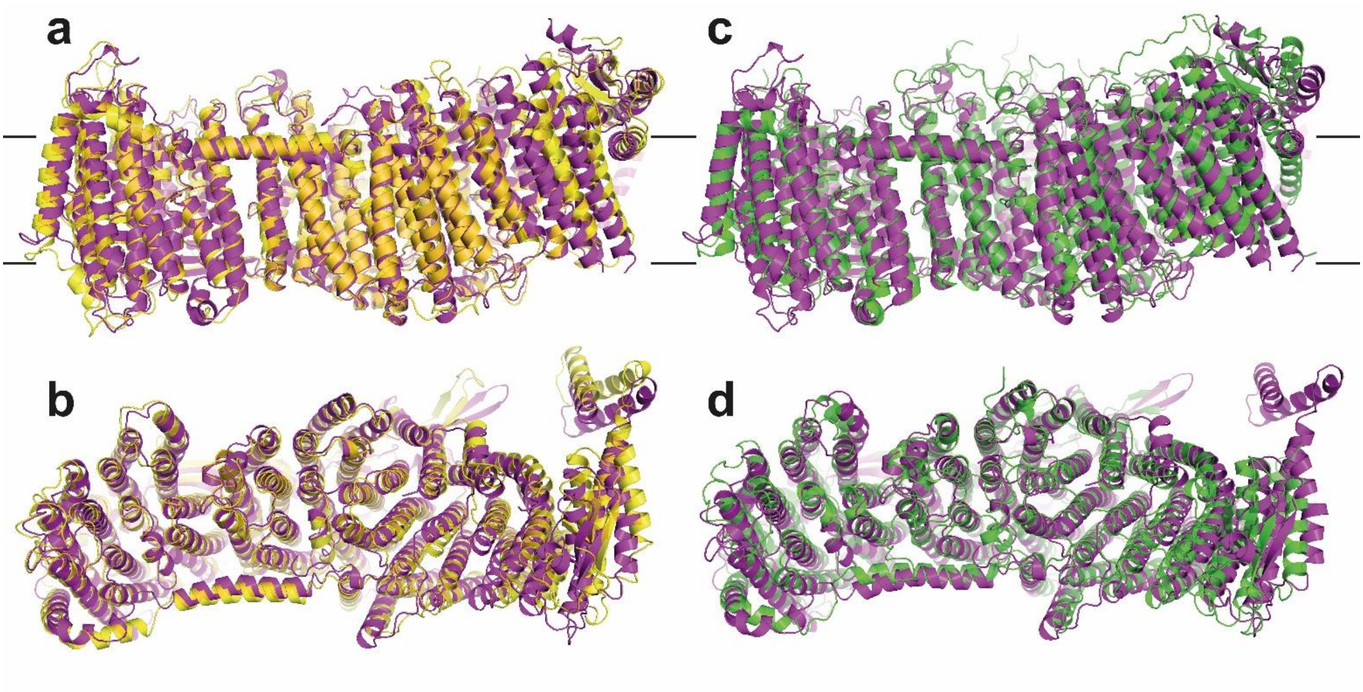

Supplementary Fig. 5.

Mrp antiporter from *B. pseudofirmus* compared with Mrp antiporters from *A. flavithermus* and *Dietzia* sp. (a) Side view and **(b)** top view of an overlay of Mrp antiporters from *B. pseudofirmus* (magenta) and *A. flavithermus* (yellow, PDB 6Z16); note that both structures show one protomer of a dimeric complex. **(c)** Side view and **(d)** top view of an overlay of Mrp antiporters from *B. pseudofirmus* (magenta) and *Dietzia* sp. (green, PDB 7D3U); note that the Mrp antiporter from *Dietzia sp.* was isolated as a monomeric complex and that MrpA and MrpB are fused.

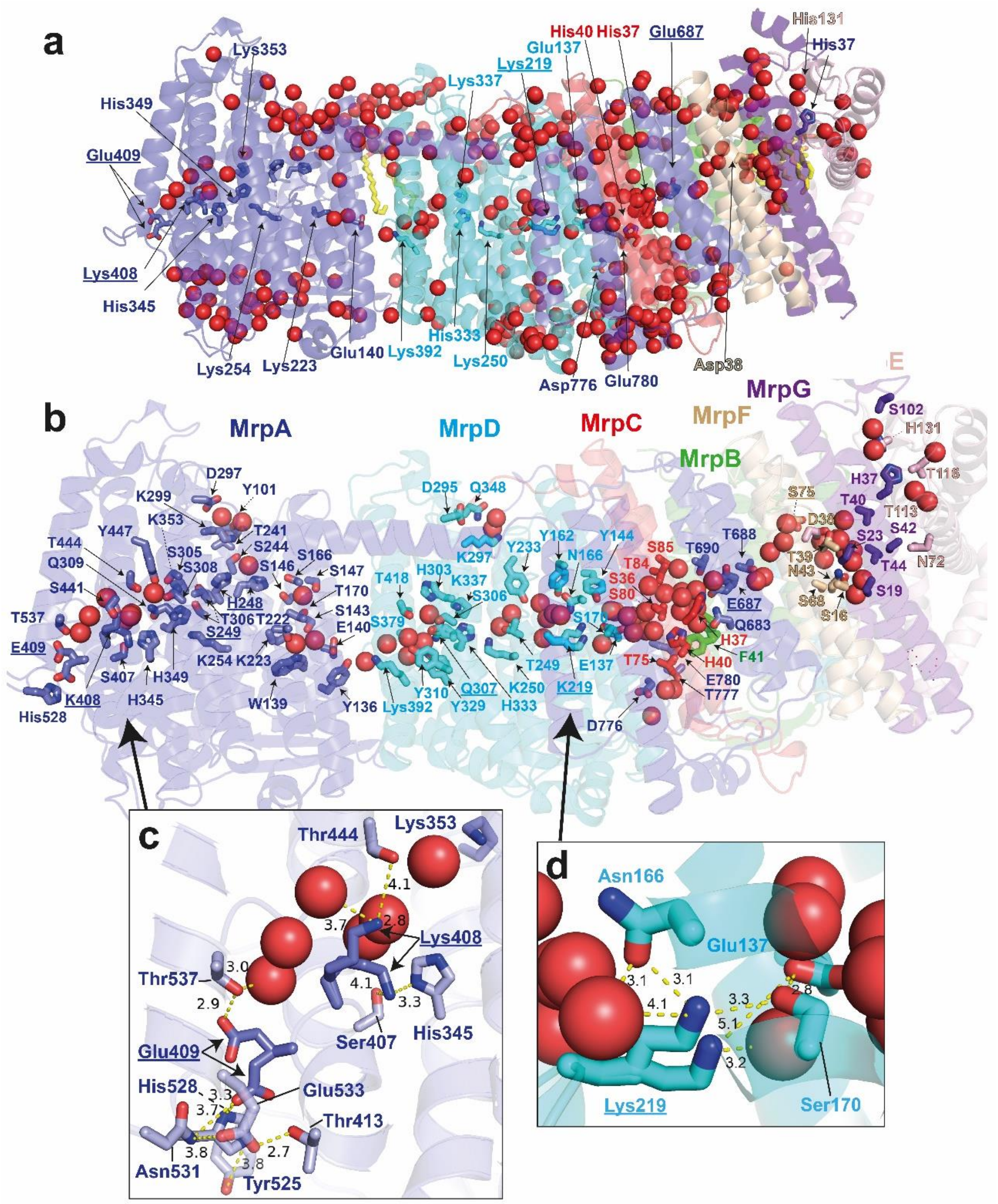

Supplementary Fig. 6.

Water molecules and ligating residues in the *B. pseudofirmus* Mrp antiporter. (a) A total of 360 water molecules (red spheres) were modelled. More than 70 water molecules define putative ion translocation pathways in the complex interior (compare Fig. 2). **(b)** Detailed view including polar residues not shown in Fig. 2. **(c)** Glu409^{MrpA} interacts with strictly conserved His528^{MrpA} and engages in a hydrogen bonding network at the periplasmic proton entry site; in a second conformation it points towards Thr537^{MrpA} and the water chain leading to His248^{MrpA}. Lys408^{MrpA} changes between a position pointing towards highly conserved Ser407^{MrpA} and His345^{MrpA} and a position where it binds two water molecules. **(d)** Lys219^{MrpD} either orients towards waters in the center of MrpD or to a water molecule that is liganded by Ser170^{MrpD} and Glu137^{MrpD} of the MrpD K/E pair.

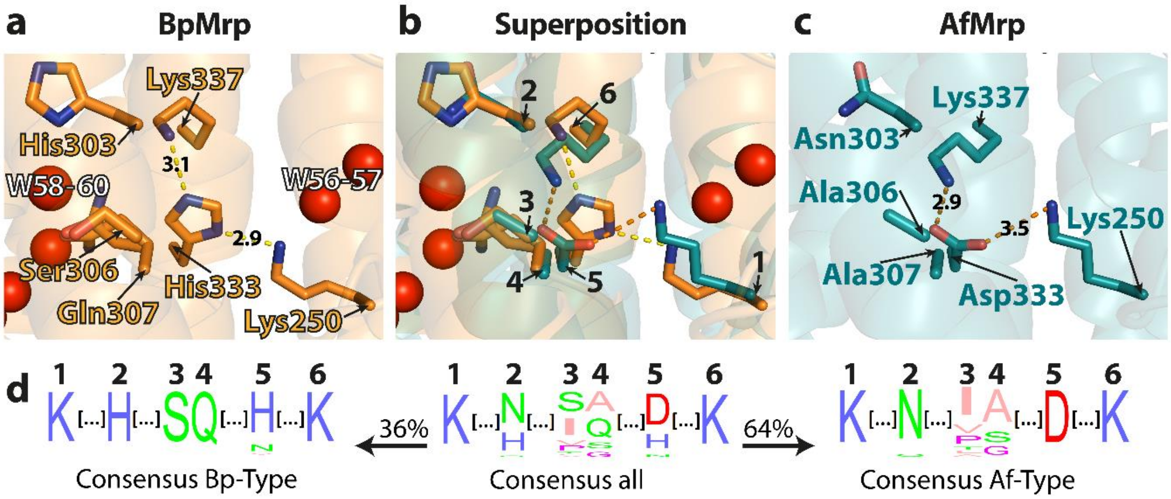

Supplementary Fig. 7.

Co-evolution of residues in MrpD of Group 1 Mrp antiporter operons. **(a)** Close-up view of the region around the strictly conserved Lys250 in TMH8 of MrpD of *B. pseudofirmus* (BpMrp) (compare Fig. 2). **(b)** Superposition of (a) and (c). **(c)** Close-up view on the region around the strictly conserved Lys250 in TMH8 of MrpD of *Anoxybacillus flavithermus* (AfMrp; PDB: 6Z16). **(d)** Consensus of 1200 sequences of Mrp group 1 operons for the residues shown in (b). Two clear subgroups can be identified. The Bp-Type (present in about 1/3 of the sequences) has His in position 2, Ser in 3, Gln in 4 and His or Asn in 5. The Af-Type (present in about 2/3 of the sequences) expresses Asn or Gln in position 2, varying residues in 3, small residues in 4 and Asp in 5.

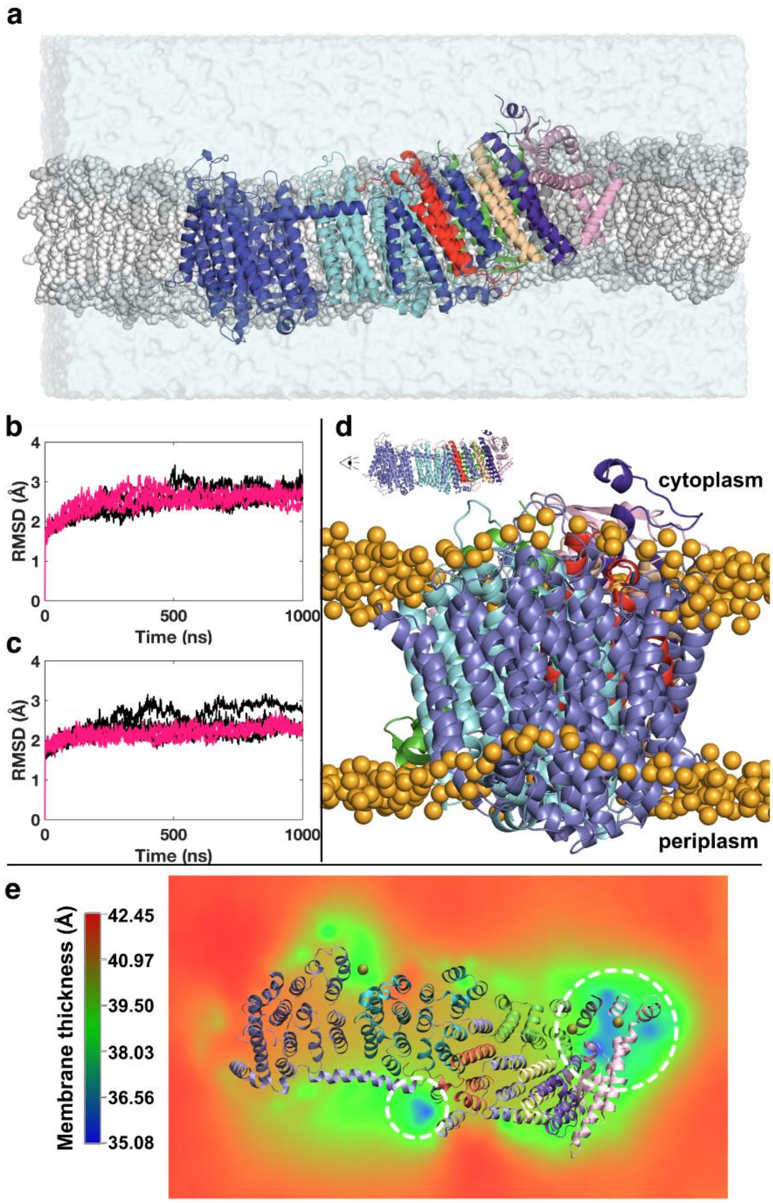

Supplementary Fig. 8.

MD simulation model systems and lipid bilayer arrangement. (a) Model system of Mrp antiporter (cartoon representation) embedded in a hybrid lipid membrane (white and gray spheres) and solvent (turquoise surface representation, Na^+ and Cl^- ions omitted for clarity). POPE lipids are shown as white and POPG as gray spheres. Protein is colored as in Figure 1. **(b)** RMSD of protein CA atoms in SA1 (black) and SB1 (pink) simulations. **(c)** RMSD of protein CA atoms in PA1 (black) and PB1 (pink) simulations. **(d)** Simulation snapshot reveals bending in the lipid membrane occurs closer to the periplasmic surface of the putative proton-uptake site. Protein is shown in ribbons representation colored as in Figure 1. Phosphorus atoms of lipids surrounding the protein are shown as yellow spheres. Lipid fatty acid side chains are omitted for clarity. **(e)** Membrane thickness observed from simulation replicas PB1 (combined 3 µs data) using VMD MEMBPLUGIN tool [10]. The thinner membrane regions are marked with white dashed circles. The protein is shown in a top view from the cytoplasmic side. The phosphorus atoms of the structural lipids are shown in olive green.

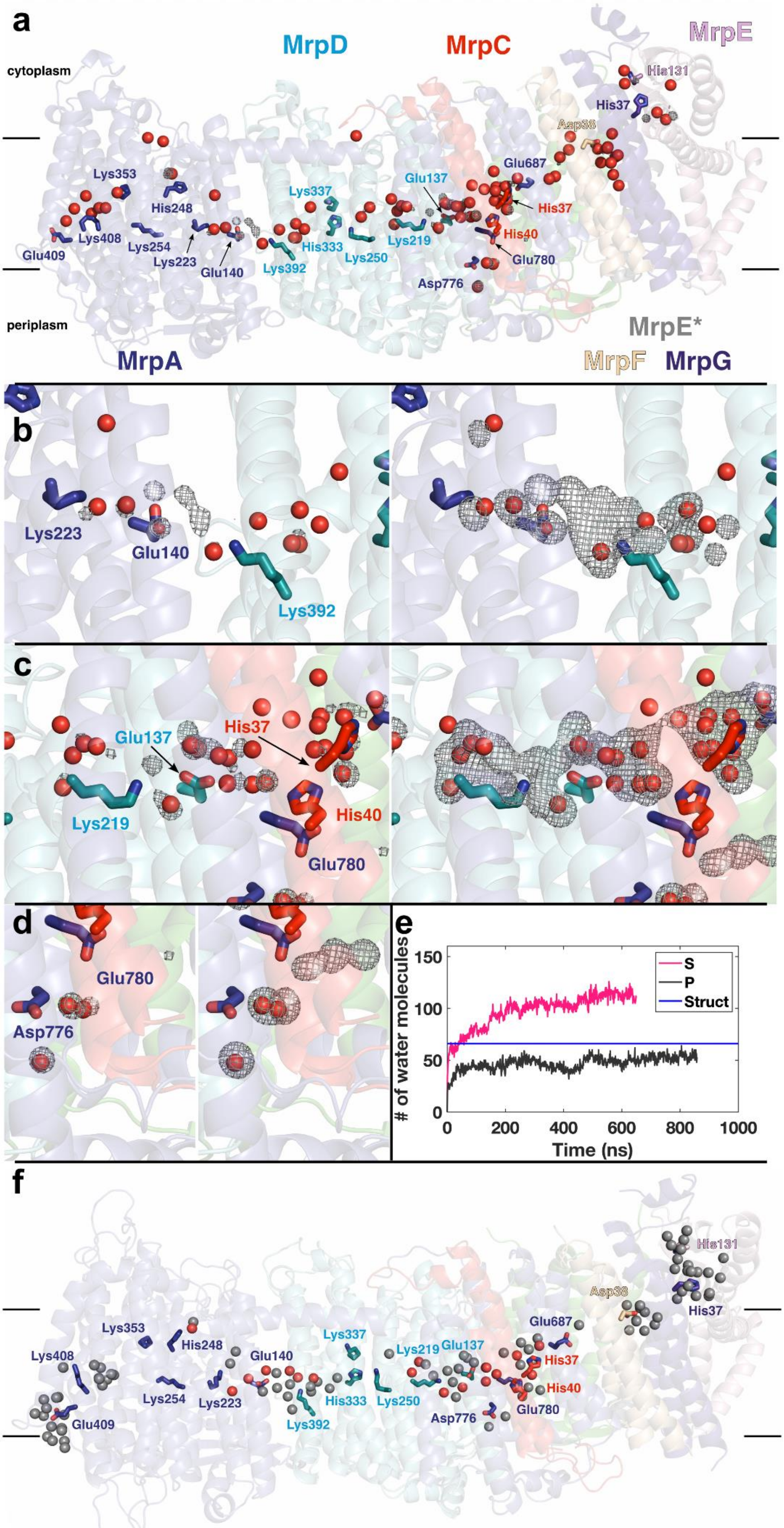

a
MrpD
MrpC
MrpE
cytoplasm
periplasm
MrpE*
MrpF
MrpG
MrpA
His131
His37
Asp38
Glu687
Lys353
His248
Lys337
Glu137
His37
His40
Glu780
Asp776
Glu409
Lys408
Lys254
Lys223
Glu140
Lys392
His333
Lys250
Lys219
b
Lys223
Glu140
Lys392
c
Glu137
His37
Lys219
His40
Glu780
d
Glu780
Asp776
e
# of water molecules
Time (ns)
S
P
Struct
0
50
100
150
200
400
600
800
1000
f

Supplementary Fig. 9.

Comparison of internal hydration volume maps at 20 and 50 % occupancies. (a) Using the same criterion as in Figure 4 (waters within 6 Å of residues displayed), the volume map of the internal hydration is shown from all PB1 simulation replicas with dark gray mesh at 50 % isovalue. Internal structural waters are shown in red spheres. **(b)** Comparison of the volume map in panel A (50%, left panel) and Figure 4a (20 %, right panel) at the interface of subunits MrpA and MrpD. **(c)** Same comparison as panel (b) but at the central axis region. **(d)** Same comparison as panel (b) but at the Glu780 region. **(e)** Number of internal water molecules in simulation states where structural water molecules were removed i.e. dry run. S refers to standard protonation state and P to propka based protonation state. The blue line indicates the number of structural internal water molecules. **(f)** Water molecules in PB1 simulation at 1000 ns. Red spheres show the internal structural waters and gray spheres the water molecules that have diffused into the interior from the bulk aqueous phase. A comparison of the hydration pattern to panel (a) and Figure 4a shows that the majority of the original internal structural waters exchange with waters from the bulk.

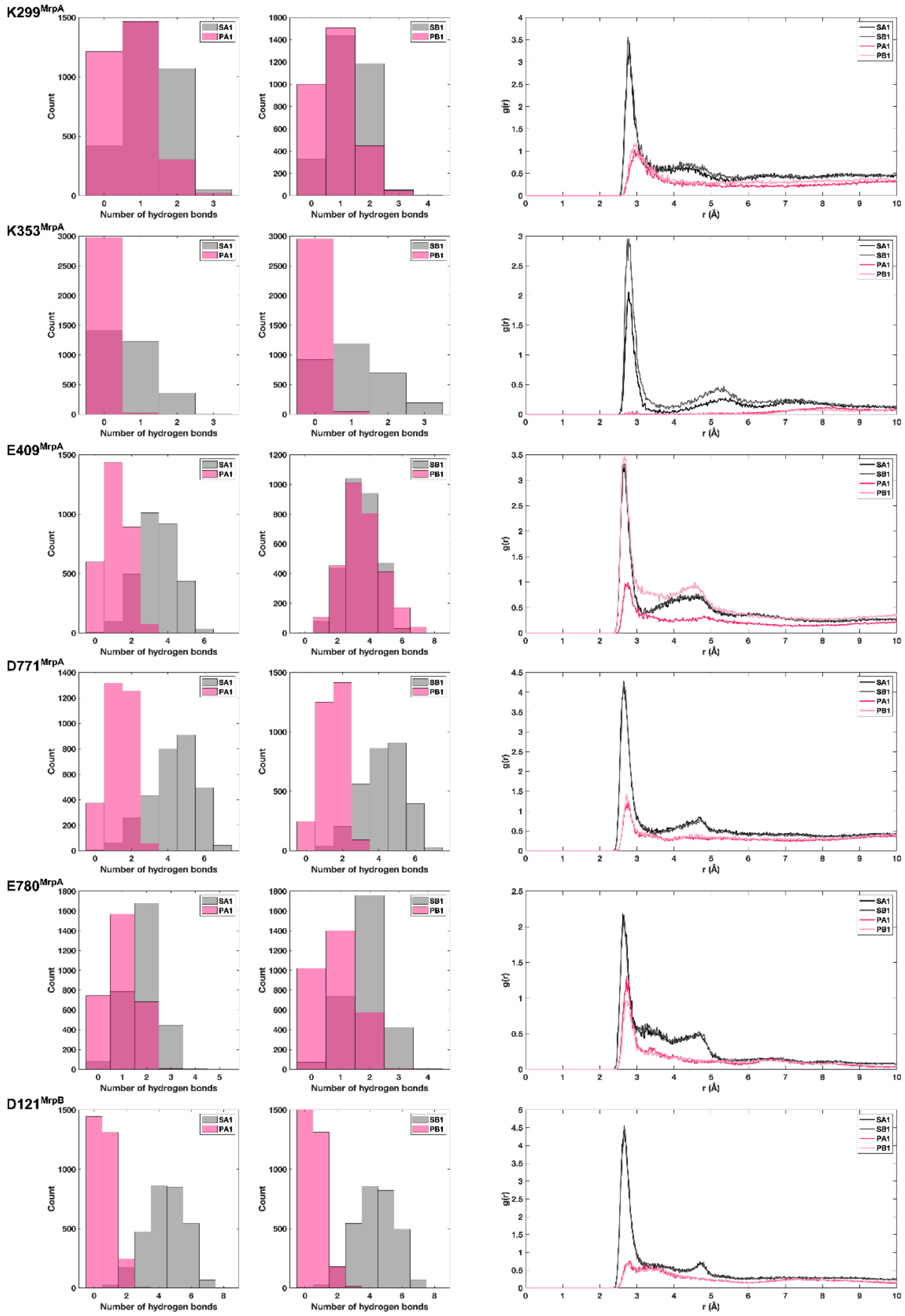

Supplementary Fig. 10.

Hydrogen bonds and radial distribution function of water molecules around selected residues in different protonation states. The histograms display the occupancy of hydrogen bond between a residue and water molecules in different protonation states (PA1, PB1, SA1 and SB1, refers to data from setups PA1, PB1, SA1 and SB1, respectively, see also Supplementary Table 6). The hydrogen bonding criteria used are Donor-Acceptor distance 3.5 Å and Donor-Hydrogen-Acceptor angle 30 degrees. The radial distribution function g(r) is calculated based on distance between sidechain nitrogen/oxygen atom and oxygen of water molecules. Overall, the trend is that the neutralization of amino acid sidechain destabilizes hydrogen bonding with water molecules.

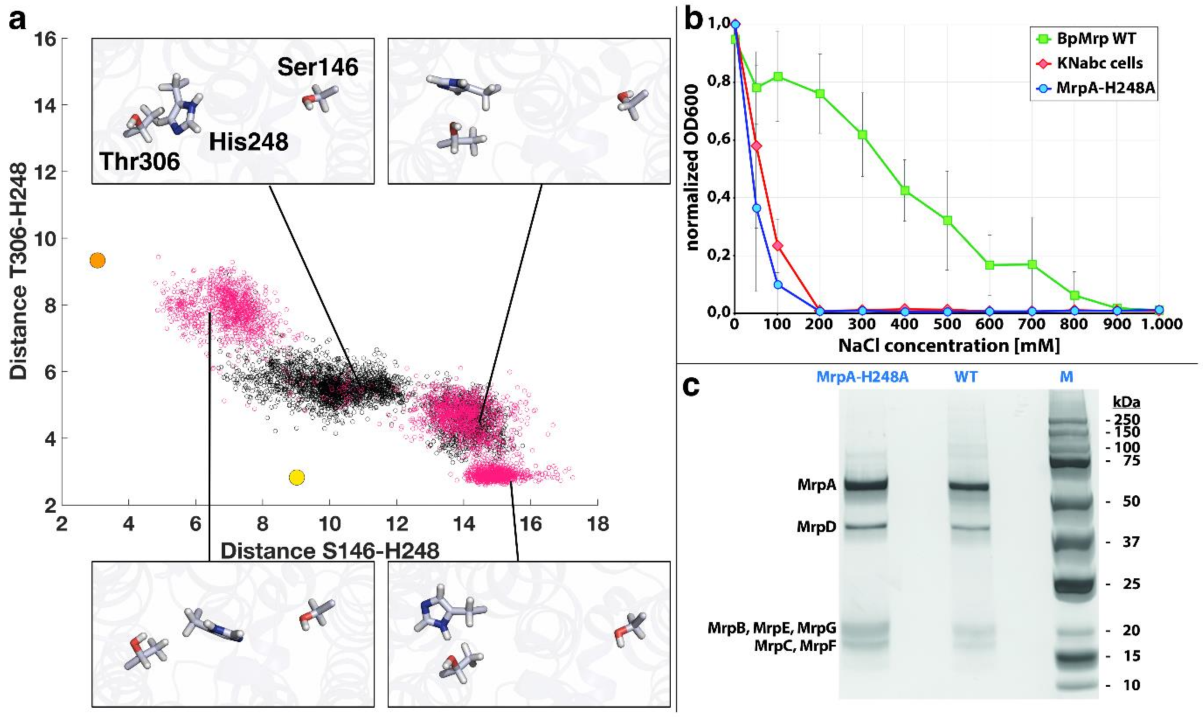

Supplementary Fig. 11.

His248^{MrpA} dynamics and mutation. **(a)** Variation in sidechain of neutral His248^{MrpA} (with δ nitrogen protonated) in S state simulations where all amino acids are in their standard states. The scatter plot shows distances between NE2 atom of His248^{MrpA} and OG atoms of Ser146/Thr306^{MrpA} from SA (pink dots) or SB (black dots) simulations. Solid yellow and orange spheres indicate the structural distances seen in alternative *A* and *B* conformations. Note that in the S state simulations, conformations of His248^{MrpA} are not populated (no overlap with solid orange and yellow circles), whereas they are clearly observed in P state simulations, where protonation states of sidechains are determined by pKa calculations (Fig. 5a). **(b)** Complementation of *E. coli* strain KNabc without intrinsic antiport activity (red curve) with an expression vector carrying the wild-type Mrp operon of *B. pseudofirmus* sustains salt tolerant growth (green curve) in contrast to complementation with the His248^{MrpA} mutant (blue curve); each data point represents the averaged measurements of at least three biological replicates with the standard deviation given as error bar. **(c)** SDS-PAGE (n=2) of purified His248^{MrpA} mutant (left) and wild-type BpMrp (middle) and molecular mass standard (right). Source data for panel (b) and (c) are provided as a Source Data file.

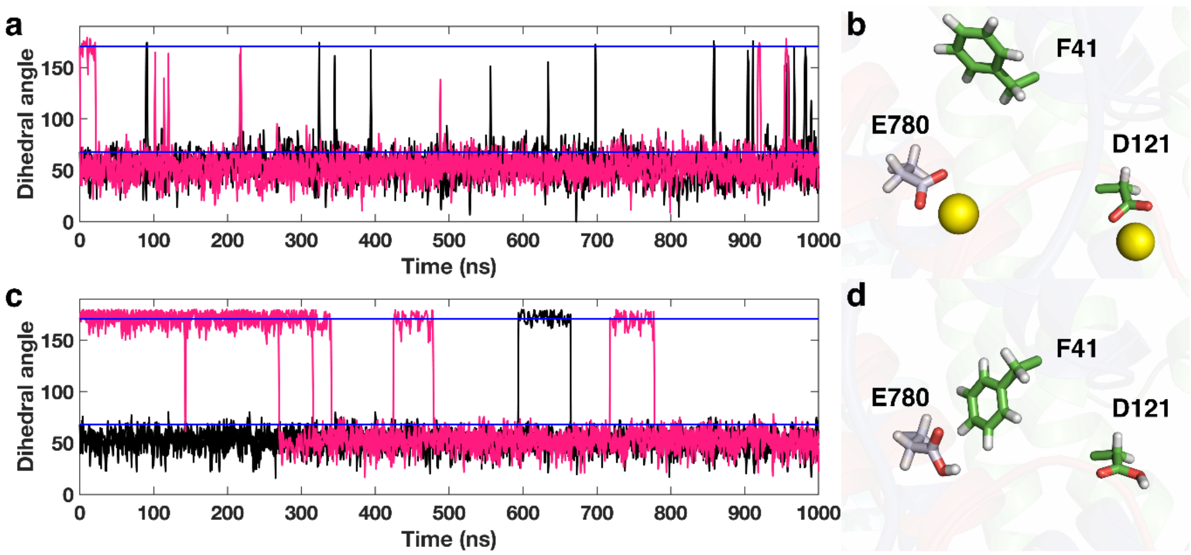

Supplementary Fig. 12.

Phe41^{MrpB} dynamics and sodium gating. (a) Dihedral angle (C-CA-CB-CD) of Phe41^{MrpB} from SA1 (3 x black) and SB1 (3 x pink) simulations. **(b)** Snapshot at 200 ns from SA1 simulation. Carbon atoms in the MrpB subunit are green; in MrpA they are lilac. In the simulations, two sodium atoms bind to residues in their charged residues spontaneously. Phe41^{MrpB} adopts the *A* conformation regardless of the initial structure as is also seen in panel (a). **(c)** Dihedral angle (C-CA-CB-CD) of Phe41^{MrpB} from PA1 (3 x black) and PB1 (3 x pink) simulations. **(d)** Snapshot at 200 ns from one PB1 simulation. Colors as in (b). Since Glu780^{MrpA} and Asp121^{MrpB} are uncharged in the PB1 simulation, no sodium ions interact with them, allowing Phe41 to remain in the *B* conformation.

Supplementary Table 1. Cryo-EM data and model statistics

	B. pseudofirmus Mrp	
**Data collection**		
Microscope	Titan Krios 2	
Camera	K3	
Magnification	105,000	
Voltage (kV)	300	
Energy filter slit width (eV)	20	
Electron flux rate (e^{-}/pixel/sec)	15.0	
Number of frames	45	
Electron exposure (e^{-}/Å^2)	50.0	
Defocus range (µm)	−0.8 to −2.0	
Calibrated pixel size (Å)	0.837	
Number of movies	7,332	
	Monomer	Dimer
**Data processing**		
Final particle images (no.)	513,743	96,337
Final pixel size (Å)	1.07136	1.07136
Symmetry imposed	C1	C2
Map resolution (Å)		
Half map FSC = 0.143	2.24	2.96
Map sharpening *B* factor (Å^2)	–16.7	–34.5
Local resolution range (Å)	2.2–2.8	
**Refinement**		
Initial model (PDB codes)	6Z16	
Refinement resolution (Å)	2.20	
Model resolution (Å)		
Map-model FSC = 0.5	2.24	
Model composition		
Non-hydrogen atoms	16,100	
Protein residues	1,957	
Water	360	
Other ligands	3 (POPE)	
Average *B* factors (Å^2)		
Protein	50.1	
Water	54.6	
Other ligands	112.6	
R.m.s. deviation		
Bond lengths (Å)	0.019	
Bond angles (°)	2.312	
**Validation**		
MolProbity score	1.79	
Clashscore	6.52	
Rotamer outliers (%)	2.50	
Cβ outliers (%)	0.49	
CaBLAM outliers (%)	0.83	
Ramachandran plot		
Favored (%)	97.37	
Allowed (%)	2.58	
Outliers (%)	0.05	

Supplementary Table 2. Identifier for water molecules in the hydrophobic transmembrane region.

water	water in PDB file		water	water in PDB file	
	Chain ID	Residue Nr.		Chain ID	Residue Nr.
**1**	G	204	**40**	B	213
**2**	G	201	**41**	B	201
**3**	E	309	**42**	A	1016
**4**	G	208	**43**	B	218
**5**	E	307	**44**	B	209
**6**	G	210	**45**	A	1055
**7**	F	115	**46**	C	220
**8**	F	108	**47**	A	1123
**9**	G	202	**48**	D	601
**10**	F	104	**49**	D	576
**11**	G	207	**50**	D	594
**12**	G	214	**51**	D	593
**13**	G	212	**52**	D	554
**14**	F	111	**53**	D	604
**15**	F	105	**54**	D	527
**16**	F	110	**55**	D	512
**17**	F	112	**56**	D	550
**18**	F	119	**57**	D	502
**19**	A	1135	**58**	D	552
**20**	A	1108	**59**	D	546
**21**	A	1018	**60**	D	605
**22**	A	1044	**61**	D	511
**23**	C	231	**62**	D	578
**24**	C	235	**63**	D	609
**25**	C	203	**64**	D	508
**26**	A	1121	**65**	A	1061
**27**	C	213	**66**	A	1063
**28**	C	233	**67**	A	1088
**29**	C	208	**68**	A	1039
**30**	C	224	**69**	A	1004
**31**	C	228	**70**	A	1117
**32**	C	212	**71**	A	1096
**33**	D	537	**72**	A	1012
**34**	D	563	**73**	A	1007
**35**	C	229	**74**	A	1086
**36**	C	216	**75**	A	1109
**37**	D	597	**76**	A	1110
**38**	A	1009	**77**	A	1136
**39**	B	217	**78**	A	1122

Supplementary Table 3. Orthologous subunits in Mrp antiporters, MBH, MBS and respiratory complex I

<table>
<tr><th>Mrp</th><th>MBH</th><th>MBS</th><th>Complex I (mitochondrial)</th><th>Complex I (bacterial)</th></tr>
<tr><td>MrpA (TM 1-14)</td><td>-</td><td rowspan="2">MbsH’</td><td rowspan="2">ND5</td><td rowspan="2">Nqo12</td></tr>
<tr><td>MrpA (TM 15,16 + lateral helix)</td><td>MbhI (C-terminal)</td></tr>
<tr><td>MrpA (TM 17-19)</td><td>MbhD</td><td>MbsD</td><td rowspan="2">ND6</td><td rowspan="2">Nqo10</td></tr>
<tr><td>MrpA (TM 20-21)</td><td>MbhE</td><td rowspan="2">MbsE</td></tr>
<tr><td>MrpB</td><td>MbhF</td><td>-</td><td>-</td></tr>
<tr><td>MrpC</td><td>MbhG</td><td>MbsG</td><td>ND4L</td><td>Nqo11</td></tr>
<tr><td>MrpD</td><td>MbhH</td><td>MbsH</td><td>ND2 / ND4</td><td>Nqo13 / Nqo14</td></tr>
<tr><td>MrpE</td><td>MbhA</td><td>MbsA</td><td>-</td><td>-</td></tr>
<tr><td>MrpF</td><td>MbhB</td><td>MbsB</td><td>-</td><td>-</td></tr>
<tr><td>MrpG</td><td>MbhC</td><td>MbsC</td><td>-</td><td>-</td></tr>
</table>

Supplementary Table 4. Conservation of residues in putative ion translocation pathways

Mrp-Subunit	Residue[a]	Conservation in Mrp [b]	Conservation in Complex I [b] (Substitutions)		Mutants with no or minor impact on activity [c]	Mutants with negative impact on activity [c]
			ND2	ND4		
MrpD	Glu137	strictly	strictly	strictly	E137D[2,5]	E137A[2,5,9]; E137Q[2,5]
MrpD	Met139	moderately (Met or Leu)	No	No	-	-
MrpD	Ser143	strictly	No	No	-	-
MrpD	Tyr162	highly	strictly	No	-	-
MrpD	Gln166	moderately	No	No (strictly Thr)	-	-
MrpD	Ser170	moderately	moderately	highly	-	-
MrpD	Leu172	No	No	No	-	-
MrpD	Met218	No	No (moderately Val or Leu)	No (moderately Phe or Met)	-	-
MrpD	Lys219	strictly	strictly	strictly	-	K219A[2]; K220A[9]
MrpD	Tyr233	strictly	moderately	No (highly His)	-	-
MrpD	Thr249	highly	No	No (highly Leu)	-	-
MrpD	Lys250	strictly	strictly	strictly	-	K251A[9]
MrpD	Asp295	No	No	highly	-	-
MrpD	Lys297	moderately (Lys or Arg)	highly	strictly	-	-
MrpD	His303	highly* (Asn or His)	No (highly Ser)	No (highly Ser)	-	-
MrpD	Ser306	moderately* (Ser or No)	No	moderately (Ala or Ser)	-	-
MrpD	Gln307	moderately* (Gln or small)	No (highly His or Asn)	No (highly His)	-	-
MrpD	Tyr328	moderately (Phe or Tyr)	No	No	-	-
MrpD	Tyr329	highly	moderately	highly	-	-
MrpD	His332	highly	No	No	-	-
MrpD	His333	highly* (Asp or His)	No (highly Tyr)	highly	-	-
MrpD	Lys337	highly*	No (moderately Asn or Thr)	No (moderately Ser or Thr)	-	-
MrpD	Phe341	highly (Phe or Tyr)	highly	highly	-	F341A[3]
MrpD	Lys392	strictly	strictly	No (strictly Glu)	-	K392A[9]
MrpD	Phe393	No	No	No	-	-
MrpD	Met423	No	No (moderately Ile, Val or Leu)	No (moderately Leu or Ile)	-	-

Mrp-Subunit	Residue[a]	Conservation in Mrp[b]	Conservation in Complex I[b] (Substitutions)	Mutants with no or minor impact on activity[c]	Mutants with negative impact on activity[c]
MrpA	Tyr101	strictly	highly	-	-
MrpA	Met118	No	No	-	-
MrpA	Phe119	strictly	highly	-	-
MrpA	Tyr136	No	No (highly Phe)	Y136A[2]	-
MrpA	Trp139	strictly	strictly	-	-
MrpA	Glu140	strictly	strictly	E113Q[6]	E113Q[5]; E132A[9]; E140A[2]
MrpA	Ser143	highly	No (highly Gly)	-	-
MrpA	Ser146	strictly	highly	-	-
MrpA	Ser147	No	No	-	-
MrpA	Met167	No	No	-	-
MrpA	Thr170	strictly	No (strictly Asn)	-	-
MrpA	Thr222	highly	No (highly Gly)	-	-
MrpA	Lys223	strictly	strictly	K196A[6]	K223A[2]; E213A[9]
MrpA	Pro240	highly	strictly	-	-
MrpA	Thr241	highly	strictly	-	-
MrpA	Pro242	strictly	strictly	-	-
MrpA	Val243	highly	highly	-	-
MrpA	Ser244	strictly	strictly	-	-
MrpA	Ala245	highly	highly	-	-
MrpA	Tyr246	highly	No (highly Leu)	-	-
MrpA	Leu247	strictly	moderately	-	-
MrpA	His248	strictly	strictly	H221A[6]	H248A[10]
MrpA	Ser249	highly	No (highly Ala)	-	-
MrpA	Ala250	strictly	moderately	-	ΔA240[9]
MrpA	Thr251	highly	highly	T224A[6]	-
MrpA	Met252	highly	highly	M225I[6]	-
MrpA	Lys254	strictly	No (moderately Thr)	K244A[9]	-
MrpA	Asp297	highly	strictly	-	-
MrpA	Lys299	strictly	strictly	K299A[2]	-
MrpA	Thr306	strictly	strictly	-	-
MrpA	Ser308	highly	strictly	-	-
MrpA	Gln309	moderately (Gln or His)	highly	-	-
MrpA	His345	highly	strictly	H345A[2]	-
MrpA	Leu346	moderately (Leu or Ile)	moderately (Leu or Val)	-	-
MrpA	His349	strictly	strictly	-	-
MrpA	Lys353	strictly	strictly	K329A[9]	-
MrpA	Ser407	highly	highly	-	-
MrpA	Lys408	strictly	strictly	-	K384A[9]
MrpA	Glu409	strictly	strictly	-	E385A[9]
MrpA	Thr413	No	No	-	-
MrpA	Thr444	moderately	highly	-	-
MrpA	Tyr447	highly	strictly	-	-
MrpA	Tyr525	No	No	-	-

Mrp-Subunit	Residue[a]	Conservation in Mrp [b]	Conservation in Complex I [b] (Substitutions)	Mutants with no or minor impact on activity [c]	Mutants with negative impact on activity [c]
MrpA	His528	highly	No	-	-
MrpA	Asn531	No	No	-	-
MrpA	Glu533	No	No	-	-
MrpA	Thr537	moderately (Thr or Ser)	No (moderately Lys or Glu)	-	-
MrpA	Ile650	No	No	-	-
MrpA	Ala656	No	No	-	-
MrpA	Val657	No	No	-	-
MrpA	Val660	No	No	-	-
MrpA	Asp678	strictly	No	D647A[9]	-
MrpA	Thr682	strictly	No	T683A[3]	-
MrpA	Gln683	strictly	No	-	-
MrpA	Val686	moderately	No	-	-
MrpA	Glu687	strictly	No	E657D[5]	E687A[3]; E656A[9]
MrpA	Thr688	No	No	-	-
MrpA	Thr690	moderately (Ser or Thr)	No	-	-
MrpA	Val691	No	No	-	-
MrpA	Leu694	No	No	-	-
MrpA	Leu704	No	No	-	-
MrpA	Glu707	No	No	-	-
MrpA	Asn766	strictly	No	-	-
MrpA	Asp771	highly	No	D736A[9]	-
MrpA	Asp776	strictly	No	D743E[5]	D743N[5]; D741A[9]
MrpA	Thr777	strictly	No	-	-
MrpA	Glu780	strictly	No	-	E747Q[4,5]; E780A[3]; E747A[4]; E745A[9]; E747D[4,5]
MrpB	His34	strictly	-	H34A[2]	-
MrpB	Pro37	strictly	-	-	P37G[2]
MrpB	Gly38	strictly	-	-	-
MrpB	Gly39	strictly	-	-	-
MrpB	Gly40	strictly	-	-	-
MrpB	Phe41	strictly	-	-	F41A[2]
MrpB	Glu111	No	-	-	-
MrpB	Glu113	No	-	-	-
MrpB	Asp121	highly	-	D121E[5]	D121A[5]; D121N[5]
MrpC	Leu26	No	No	-	-
MrpC	Arg27	moderately (Arg, Gln, or Lys)	No	-	-
MrpC	Val30	No	No	-	-
MrpC	Gly31	highly	No	-	-
MrpC	Ser36	moderately	No	-	-
MrpC	His37	moderately (His, Tyr or Asn)	No	-	-
MrpC	His40	moderately (His or Asn)	No	-	-

Mrp-Subunit	Residue[a]	Conservation in Mrp [b]	Conservation in Complex I [b] (Substitutions)	Mutants with no or minor impact on activity [c]	Mutants with negative impact on activity [c]
MrpC	Leu74	strictly	No	-	-
MrpC	Thr75	strictly	No (highly Ala)	-	T75A[2]
MrpC	Ala76	moderately (Ala or Ser)	moderately	-	-
MrpC	Ile77	strictly	No	-	I76F[7]
MrpC	Val78	strictly	No (highly Glu)	-	-
MrpC	Ile79	strictly	No	-	-
MrpC	Ser80	No	No	-	-
MrpC	Thr84	moderately	No	-	-
MrpC	Ser85	No	No	-	-
MrpE	Thr113	highly	-	-	T113A[1,2]; T113Y[2]
MrpE	Thr116	moderately (Thr or Ser)	-	-	-
MrpE	<u>Met119</u>	No	-	-	-
MrpE	His131	strictly	-	H131A[1]	-
MrpF	<u>Met13</u>	No	-	-	-
MrpF	<u>Ile34</u>	Moderately (Val or Ile)	-	-	-
MrpF	Asp38	highly	-	D36A/F40D[9]; D38N[5]; D36A/I33D[9]	D38A[5]; D36A[9]; D35L[8]; D36L[9]; D36N[9]; D38E[5]
MrpF	Thr39	No	-	-	-
MrpF	Asn43	No	-	-	-
MrpF	Ser68	No	-		
MrpF	<u>Ser75</u>	Moderately Thr	-	-	-
MrpG	Ser19	No	-	-	-
MrpG	Ser23	No	-	-	-
MrpG	His37	Moderately	-	-	-
MrpG	Thr40	moderately (Thr or Ser)	-	-	-
MrpG	Thr44	moderately (Thr or Ser)	-	-	-
MrpG	<u>Phe75</u>	highly	-	-	-
MrpG	Thr79	highly	-	-	-

[a] Residues that show multiple conformations in our structure are underlined.

[b] The conservation is given in four categories based on alignments of 1200-2000 non-redundant sequences: strict (>99.5%); high (>90%); moderate (>80% or >90% in case residues with similar properties are conserved at this position) or not conserved (deviating from BpMrp).

[c] Mutations are given in short form with the sequence numbering from the original source and the source given in superscript: [1] ref [11]; [2] ref [12]; [3] ref [13]; [4] ref [14]; [5] ref [15]; [6] ref [16]; [7] ref [17]; [8] ref [18]; [9] ref [19]; [10] this work

* Refers to group 1 operons (separate MrpA and MrpB) only. See Supplementary Fig. 7

Supplementary Table 5. Occupancies of alternative *A* and *B* conformations

chain	residue type	residue number	alt_*A*	alt_*B*
A	MET	118	0,45	0,55
A	PHE	119	0,45	0,55
A	MET	167	0,45	0,55
A	TYR	246	0,50	0,50
A	LEU	247	0,43	0,57
A	HIS	248	0,43	0,57
A	SER	249	0,43	0,57
A	ALA	250	0,43	0,57
A	THR	251	0,43	0,57
A	MET	252	0,43	0,57
A	LEU	346	0,43	0,57
A	LYS	408	0,67	0,33
A	GLU	409	0,69	0,31
A	LEU	681	0,69	0,31
A	GLU	687	0,58	0,42
A	ASN	766	0,48	0,52
B	PHE	41	0,64	0,36
D	MET	139	0,47	0,53
D	LEU	172	0,47	0,53
D	MET	218	0,43	0,57
D	LYS	219	0,45	0,55
D	GLN	307	0,45	0,55
D	TYR	328	0,69	0,31
D	MET	423	0,54	0,46
E	MET	119	0,54	0,46
F	MET	13	0,58	0,42
F	ILE	34	0,76	0,24
F	SER	75	0,42	0,58
G	PHE	75	0,52	0,48

Supplementary Table 6. Model systems and simulation time scales.

System	Conformation#	Charge state	Simulation length
SA1	*A*	Standard	3 x 1000 ns
SB1	*B*	Standard	3 x 1000 ns
PA1	*A*	Propka-based	3 x 1000 ns
PB1	*B*	Propka-based	3 x 1000 ns
PBE	*B*	Propka-based except $H248^{MrpA}$ HSE*	3 x 500 ns
PBP	*B*	Propka-based except $H248^{MrpA}$ HSP**	3 x 500 ns
SMA1	*A*	Standard, H248A	500 ns
SMB1	*B*	Standard, H248A	500 ns
PMA1	*A*	Propka, H248A	500 ns
PMB1	*B*	Propka, H248A	500 ns
SNA1	*A*	Standard, sodium ion modelled near anionic $D38^{MrpF}$	800 ns
SNA2	*A*	Standard, snapshot from SNA1, but $D38^{MrpF}$ neutral	160 ns
SNA3	*A*	Standard, snapshot from SNA2, but $E687^{MrpA}$ neutral	800 ns
SNA4	*A*	Standard, snapshot from SA1 but sodium ion modelled in the hydrated path towards $E687^{MrpA}$	100 ns, 13 ns
SNA5	*A*	Standard, snapshot from SA1, except $E687^{MrpA}$, $D771^{MrpA}$, $D678^{MrpA}$, $D121^{MrpB}$, $E113^{MrpB}$ and $D38^{MrpF}$ neutral. Sodium ion modelled in between $E687^{MrpA}$ and $H37^{MrpC}$.	110 ns
SNA6	*A*	Standard, snapshot from SNA5 but $H37^{MrpC}$ and $H40^{MrpC}$ doubly-protonated.	280 ns, 110 ns
SNA7	*A*	Snapshot from SNA6, but $E137^{MrpD}$ neutral.	2 x 110 ns

# Alternative location A and B, as defined in PDB file.

* HSE means neutral histidine with ε nitrogen protonated

** HSP means that histidine is doubly-protonated

Supplementary Table 7. Protonation states of amino acids in *A* and *B* conformations based on pKa calculations (see methods)

		Conformation *A*		Conformation *B*	
**Subunit**	**Residue**	**pKa**	**Charge state**	**pKa**	**Charge state**
MrpA	Asp678	7.24	0	7.36	0
MrpA	Asp771	7.65	0	7.65	0
MrpA	Glu409	7.20	0	6.79	-1
MrpA	Glu687	8.42	0	7.75	0
MrpA	Glu780	8.54	0	9.52	0
MrpA	His470	7.17	+1	7.17	+1
MrpA	Lys223	6.94	0	6.65	0
MrpA	Lys254	5.85	0	5.90	0
MrpA	Lys299	6.86	0	6.84	0
MrpA	Lys353	6.40	0	6.43	0
MrpA	Lys408	7.94	+1	6.37	0
MrpB	Asp121	7.34	0	7.35	0
MrpD	Lys250	6.31	0	6.31	0
MrpD	Lys337	5.61	0	5.61	0
MrpF	Asp38	6.70	-1	7.03	0

Supplementary Table 8. Residence times of water molecules around key titratable residues based on hydrogen bond dynamics and lifetimes. Blue – refers to those bulk-facing residues around which the residence of water molecules does not differ much with respect to changes in conformational/protonation states. Green – change in protonation state of amino acid residue changes the residence times of water molecules around it. Dark orange – putative sodium binding site, where no major differences in residence times of water molecules are observed either when sodium ion binds or when residue is protonated. NA – residence time not available because autocorrelation did not approach to zero in the given simulation time scales or higher standard deviation of the tail (see ref. [20]). >ns – residence time is expected to be much longer than nanoseconds or tens of nanoseconds (lower bound value given). This may occur due to stable hydrogen bonding between structural water molecule and amino acid (red color). R1-R3 refer to three simulation replicas. The hydrogen bonding criteria used in this analysis is as follows: donor-acceptor distance 3.5 Å and donor-hydrogen-acceptor angle 35˚, and forward hydrogen bonding lifetimes are reported using GROMACS software. ps – picoseconds; q – charge of the sidechain; S – standard and P – Propka-based charged state (see methods); A and B are alternative conformations.

Protein Chain	Residue id	Residue type		S, A	P, A	S, B	P, B
A	771	ASP	q<br>R1<br>R2<br>R3	-1<br>271.9 ps<br>403.58 ps<br>257.19 ps	0<br>149.714 ps<br>373.78 ps<br>348.918 ps	-1<br>508.188 ps<br>193.27 ps<br>301.16 ps	0<br>408.642 ps<br>392.166 ps<br>183.7 ps
A	299	LYS	q<br>R1<br>R2<br>R3	+1<br>320.4 ps<br>347.1 ps<br>300.39 ps	0<br>635.70 ps<br>266.236 ps<br>509.57 ps	+1<br>285.14 ps<br>190.57 ps<br>340.18 ps	0<br>389.926 ps<br>632.899 ps<br>304.9 ps
B	121	ASP	q<br>R1<br>R2<br>R3	-1<br>668.27 ps<br>692.31 ps<br>529.8 ps	0<br>678.75 ps<br>650.149 ps<br>553.53 ps	-1<br>833.09 ps<br>721.618 ps<br>480.191 ps	0<br>775.316 ps<br>819.972 ps<br>647.4 ps
A	470	HIS	q<br>R1<br>R2<br>R3	0<br>7.5 ps<br>8.08 ps<br>9.627 ps	+1<br>18.860 ps<br>30.795 ps<br>20.052 ps	0<br>5.78 ps<br>6.46 ps<br>14.61 ps	+1<br>35.948 ps<br>82.866 ps<br>34.69 ps
A	409	GLU	q<br>R1<br>R2<br>R3	-1<br>313.49 ps<br>320.58 ps<br>1.47 ns	0<br>1.33 ns<br>1.222 ns<br>NA	-1<br>457.5 ps<br>528.6 ps<br>330.73 ps	-1<br>235.188 ps<br>338.500 ps<br>272.5 ps
A	408	LYS	q<br>R1<br>R2<br>R3	+1<br>425.97 ps<br>530.33 ps<br>484.40 ps	+1<br>NA (>ns)<br>NA (>ns)<br>NA (>ns)	+1<br>504.72 ps<br>553.84 ps<br>326.2 ps	0<br>NA (>ns)<br>NA (>ns)<br>NA (>ns)
A	780	GLU	q<br>R1<br>R2<br>R3	-1<br>1.64 ns<br>2.57 ns<br>1.44 ns	0<br>1.57 ns<br>NA (>ns)<br>1.68 ns	-1<br>2.58 ns<br>986.65 ps<br>1.40 ns	0<br>1.331 ns<br>1.655 ns<br>1.49 ns
A	223	LYS	q	+1<br>NA (>ns)	0<br>NA (>ns)	+1<br>NA (>ns)	0<br>NA (>ns)
A	254	LYS	q	+1<br>NA (>ns)	0<br>>ns	+1<br>NA (>ns)	0<br>>ns
A	678	ASP	q	-1<br>NA (>ns)	0<br>NA (>ns)	-1<br>NA (>ns)	0<br>NA (>ns)

Supplementary References

1. Kampjut, D. & Sazanov, L. A. The coupling mechanism of mammalian respiratory complex I. *Science (New York, N.Y.)* **370**; 10.1126/science.abc4209 (2020).
2. Parey, K. *et al.* High-resolution structure and dynamics of mitochondrial complex I-Insights into the proton pumping mechanism. *Sci Adv* **7,** eabj3221; 10.1126/sciadv.abj3221 (2021).
3. Steiner, J. & Sazanov, L. Structure and mechanism of the Mrp complex, an ancient cation/proton antiporter. *eLife* **9** (2020).
4. Yu, H. *et al.* Structure of an Ancient Respiratory System. *Cell* **173,** 1636-1649.e16; 10.1016/j.cell.2018.03.071 (2018).
5. Haapanen, O. & Sharma, V. Role of water and protein dynamics in proton pumping by respiratory complex I. *Sci Rep* **7,** 7747; 10.1038/s41598-017-07930-1 (2017).
6. Roh, S.-H. *et al.* Cryo-EM and MD infer water-mediated proton transport and autoinhibition mechanisms of Vo complex. *Sci Adv* **6**; 10.1126/sciadv.abb9605 (2020).
7. Sievers, F. *et al.* Fast, scalable generation of high-quality protein multiple sequence alignments using Clustal Omega. *Mol Sys Biol* **7,** 539; 10.1038/msb.2011.75 (2011).
8. Waterhouse, A. M., Procter, J. B., Martin, D. M. A., Clamp, M. & Barton, G. J. Jalview Version 2--a multiple sequence alignment editor and analysis workbench. *Bioinformatics* **25,** 1189–1191; 10.1093/bioinformatics/btp033 (2009).
9. Larkin, M. A. *et al.* Clustal W and Clustal X version 2.0. *Bioinformatics (Oxford, England)* **23,** 2947–2948; 10.1093/bioinformatics/btm404 (2007).
10. Guixà-González, R. *et al.* MEMBPLUGIN: studying membrane complexity in VMD. *Bioinformatics (Oxford, England)* **30,** 1478–1480; 10.1093/bioinformatics/btu037 (2014).
11. Morino, M., Natsui, S., Swartz, T. H., Krulwich, T. A. & Ito, M. Single gene deletions of mrpA to mrpG and mrpE point mutations affect activity of the Mrp Na^+/H^+ antiporter of alkaliphilic Bacillus and formation of hetero-oligomeric Mrp complexes. *J Bact* **190,** 4162–4172; 10.1128/JB.00294-08 (2008).
12. Morino, M. *et al.* Single site mutations in the hetero-oligomeric Mrp antiporter from alkaliphilic Bacillus pseudofirmus OF4 that affect Na^+/H^+ antiport activity, sodium exclusion, individual Mrp protein levels, or Mrp complex formation. *J Biol Chem* **285,** 30942–30950; 10.1074/jbc.M110.118661 (2010).
13. Morino, M., Ogoda, S., Krulwich, T. A. & Ito, M. Differences in the phenotypic effects of mutations in homologous MrpA and MrpD subunits of the multi-subunit Mrp-type Na^+/H^+ antiporter. *Extremophiles : life under extreme conditions* **21,** 51–64; 10.1007/s00792-016-0877-z (2017).
14. Kosono, S. *et al.* Functional involvement of membrane-embedded and conserved acidic residues in the ShaA subunit of the multigene-encoded Na^+/H^+ antiporter in *Bacillus subtilis*. *Biochim Biophys Acta* **1758,** 627–635; 10.1016/j.bbamem.2006.04.012 (2006).
15. Kajiyama, Y., Otagiri, M., Sekiguchi, J., Kudo, T. & Kosono, S. The MrpA, MrpB and MrpD subunits of the Mrp antiporter complex in Bacillus subtilis contain membrane-embedded and essential acidic residues. *Microbiology* **155,** 2137–2147; 10.1099/mic.0.025205-0 (2009).
16. Sperling, E., Górecki, K., Drakenberg, T. & Hägerhäll, C. Functional Differentiation of Antiporter-Like Polypeptides in Complex I; a Site-Directed Mutagenesis Study of Residues Conserved in MrpA and NuoL but Not in MrpD, NuoM, and NuoN. *PloS one* **11,** e0158972; 10.1371/journal.pone.0158972 (2016).

17. Xu, N. *et al.* The Lysine 299 Residue Endows the Multisubunit Mrp1 Antiporter with Dominant Roles in Na+ Resistance and pH Homeostasis in *Corynebacterium glutamicum*. *Appl Env Microbiol* **84**; 10.1128/AEM.00110-18 (2018).

18. Steiner, J. & Sazanov, L. Structure and mechanism of the Mrp complex, an ancient cation/proton antiporter. *eLife* **9**; 10.7554/eLife.59407 (2020).

19. Li, B. *et al.* Structure of the *Dietzia* Mrp complex reveals molecular mechanism of this giant bacterial sodium proton pump. *Proc Natl Acad Sci U S A* **117,** 31166–31176; 10.1073/pnas.2006276117 (2020).

20. van der Spoel, D., van Maaren, P. J., Larsson, P. & Tîmneanu, N. Thermodynamics of hydrogen bonding in hydrophilic and hydrophobic media. *J Phys Chem B* **110,** 4393–4398; 10.1021/jp0572535 (2006).
