# Supplementary material for: Ion transfer mechanisms in Mrp-type antiporters from high resolution cryoEM and molecular dynamics simulations
Source: Nat Commun. 2022 Oct 14;13:6091. doi: 10.1038/s41467-022-33640-y (PMC9568556; doi:10.1038/s41467-022-33640-y)
Supplement: Supplementary file 3 — Description of Additional Supplementary Files [file 41467_2022_33640_MOESM3_ESM.pdf]

File name: Supplementary Movie 1

Description: A histidine switch operates in MrpA. His248^{MrpA} is part of a mobile segment (position 246 to 252) of TMH8 near the cytoplasmic side. In the *A* conformation, His248^{MrpA} forms a hydrogen bond to Thr306^{MrpA}, which switches to Ser146^{MrpA} in the *B* conformation. Hydrated pathways link Thr306^{MrpA} to the proton entry site of MrpA at Glu409^{MrpA}/Lys408^{MrpA} (see Fig. 2). Ser146^{MrpA} is linked to the Glu140^{MrpA}/Lys223^{MrpA} pair and further to MrpD. In the *B* conformation, a water molecule (W69; not shown - see Fig. 2) bound to Ser244^{MrpA} links His248^{MrpA} to a chain of polar residues leading to the cytoplasmic side. Hydrophobic interactions of Phe119^{MrpA} with Leu247^{MrpA} and Met252^{MrpA} in the neighboring TMH4 limit the movement of the TMH8 segment. MD simulations show that movement of His248^{MrpA} depends on the protonation state.
